# Prediction of American Society of Anesthesiologists Physical Status Classification from preoperative clinical text narratives using natural language processing

**DOI:** 10.1186/s12871-023-02248-0

**Published:** 2023-09-04

**Authors:** Philip Chung, Christine T. Fong, Andrew M. Walters, Meliha Yetisgen, Vikas N. O’Reilly-Shah

**Affiliations:** 1 Department of Anesthesiology & Pain Medicine, University of Washington, 1959 NE Pacific Street, BB-1469, Box 356540, Seattle, WA 98195-6540 USA; 2 Department of Biomedical & Health Informatics, University of Washington, 850 Republican Street, Box 358047, Seattle, WA 98109 USA; 3 Department of Linguistics, University of Washington, 850 Republican Street, Box 358047, Seattle, WA 98109 USA

**Keywords:** Natural language processing, Perioperative risk, Machine learning

## Abstract

**Background:**

Electronic health records (EHR) contain large volumes of unstructured free-form text notes that richly describe a patient’s health and medical comorbidities. It is unclear if perioperative risk stratification can be performed directly from these notes without manual data extraction. We conduct a feasibility study using natural language processing (NLP) to predict the American Society of Anesthesiologists Physical Status Classification (ASA-PS) as a surrogate measure for perioperative risk. We explore prediction performance using four different model types and compare the use of different note sections versus the whole note. We use Shapley values to explain model predictions and analyze disagreement between model and human anesthesiologist predictions.

**Methods:**

Single-center retrospective cohort analysis of EHR notes from patients undergoing procedures with anesthesia care spanning all procedural specialties during a 5 year period who were not assigned ASA VI and also had a preoperative evaluation note filed within 90 days prior to the procedure. NLP models were trained for each combination of 4 models and 8 text snippets from notes. Model performance was compared using area under the receiver operating characteristic curve (AUROC) and area under the precision recall curve (AUPRC). Shapley values were used to explain model predictions. Error analysis and model explanation using Shapley values was conducted for the best performing model.

**Results:**

Final dataset includes 38,566 patients undergoing 61,503 procedures with anesthesia care. Prevalence of ASA-PS was 8.81% for ASA I, 31.4% for ASA II, 43.25% for ASA III, and 16.54% for ASA IV-V. The best performing models were the BioClinicalBERT model on the truncated note task (macro-average AUROC 0.845) and the fastText model on the full note task (macro-average AUROC 0.865). Shapley values reveal human-interpretable model predictions. Error analysis reveals that some original ASA-PS assignments may be incorrect and the model is making a reasonable prediction in these cases.

**Conclusions:**

Text classification models can accurately predict a patient’s illness severity using only free-form text descriptions of patients without any manual data extraction. They can be an additional patient safety tool in the perioperative setting and reduce manual chart review for medical billing. Shapley feature attributions produce explanations that logically support model predictions and are understandable to clinicians.

**Supplementary Information:**

The online version contains supplementary material available at 10.1186/s12871-023-02248-0.

## Background

Machine learning and natural language processing (NLP) techniques, coupled with adoption of electronic health records (EHR), and widespread availability of high-performance computational resources offer new avenues for perioperative risk stratification whereby free-form text sources, such as medical notes, may be directly loaded into prediction models without the need to define, input or abstract predetermined data elements (e.g. diagnoses, medications, etc.). This offers the opportunity to use these techniques for preoperative assessment triage, flagging of critical/pertinent data in a voluminous electronic medical record, and a variety of other use cases based on clinician notes, which often contain narratives that richly and concisely describe a nuanced clinical picture of the patient while simultaneously prioritizing the clinician’s pertinent concerns. Unlike historical keyword-based approaches, modern NLP techniques using large pretrained language models are able to account for inter-word dependencies across the entire text sequence and have been shown to achieve state of the art performance on a variety of NLP tasks [1–4] including text classification [5, 6]. However, it is unknown whether these techniques can be successfully applied to perioperative risk stratification.

In this feasibility study, we hypothesize that NLP models can be applied to unstructured anesthesia preoperative evaluation notes written by clinicians to predict the American Society of Anesthesiologists Physical Status (ASA-PS) score [7, 8]. These preoperative evaluation notes are a pertinent summary of the patient’s medical and surgical history and describe why the patient is having surgery, all of which reflect the patient’s pre-anesthesia medical comorbidities that the ASA-PS aims to represent. In particular, we investigate four different text classification approaches that span the spectrum of historical and modern techniques: (1) random forest [9] with n-gram and term frequency-inverse document frequency (TFIDF) transform [10], (2) support vector machine [11] with n-gram and TFIDF transform, (3) fastText [12, 13] word vector model, and (4) BioClinicalBERT deep neural network language model. We also investigate the impact of using the entire note versus specific note sections. We compare the model’s prediction against the ASA-PS assigned by the anesthesiologist on the day of surgery and assess catastrophic errors made by one of these models. Finally, we use Shapley values to visualize which sections of note text were associated with the model’s predictions to explain these catastrophic errors. This approach shows that it is possible for clinicians to understand how complex NLP models are making their predictions, which is an important criteria for clinical adoption.

## Methods

**Fig. 1:**
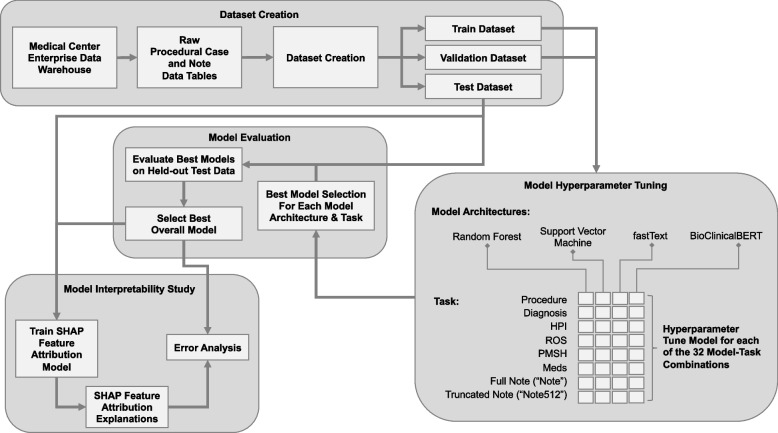
Flowchart of study design: dataset creation, model development, evaluation, and interpretation

This retrospective study of routinely collected health records data was approved by the University of Washington Institutional Review Board with a waiver of consent. This study followed the Transparent Reporting of a Multivariable Prediction Model for Individual Prognosis or Diagnosis (TRIPOD) guideline [14] and other guidelines specific to machine learning projects [15–17]. Figure 1 depicts a flow diagram of study design.

### Study cohort

Inclusion criteria were patients who had a procedure with anesthesia at the University of Washington Medical Center or Harborview Medical Center from January 1, 2016 – March 29, 2021 where the patient also had an anesthesia preoperative evaluation note filed up to 6 h after the anesthesia end time. This 6-h grace period reflects the reality that in some urgent or emergency situations or due to EHR behavior, text documentation may be time stamped out of order.

The anesthesia preoperative evaluation note must have contained the following sections: History of Present Illness (HPI), Past Medical and Surgical History (PMSH), Review of Systems (ROS), and Medications; notes missing at least one of these sections were excluded. No other note type was used. Cases must have had a recorded value for ASA-PS assigned by the anesthesiologist of record, a free-form text Procedure description, and a free-form text Diagnosis description; cases missing at least one of these values are excluded.

A unit of analysis is defined as a single case with an anesthesia preoperative evaluation note filed within 90 days of the procedure. This unit was chosen because ASA-PS is typically recorded on a per-case basis by the anesthesiologist to reflect the patient’s pre-anesthesia medical comorbidities at the time of the procedure. Likewise, preoperative evaluation notes filed > 90 days before the case may not reflect the patient’s current state of health, so are excluded. Data was randomly split 70%-10%-20% into training, validation, and test datasets respectively. Patients with multiple cases were randomized into a single data split to avoid information leakage between the three datasets. New case number identifiers were generated for this study and used to refer to each case.

### Outcomes

The outcome variable is a modified ASA-PS with valid values of ASA I, ASA II, ASA III, ASA IV-V. ASA V cases are extremely rare, resulting in class imbalances that affect model training and performance. Thus ASA IV and V were combined into a compound class “IV-V”. ASA VI organ procurement cases are excluded. The final categories retain the spirit of the ASA-PS for perioperative risk stratification and resembles the original ASA-PS devised by Saklad in 1941 [7, 18]. The emergency surgery modifier “E” was discarded.

### Predictors and data preparation

Free-form text from the anesthesia preoperative evaluation note is organized into many sections. Regular expressions are used to extract HPI, PMSH, ROS, and medications from the note. While diagnosis and procedure sections exist within the note, they were less frequently documented than in the procedural case booking data from the surgeon. Therefore, free-form text for these sections were taken from the case booking. Newline characters and whitespaces were removed from the text. Note section headers were excluded so that only the body of text from each section is included. We used text from each section to train models for ASA-PS prediction, resulting in 8 prediction tasks: Diagnosis, Procedure, HPI, PMSH, ROS, Medications (Meds), Note, Truncated Note (Note512). “Note” refers to using the whole note text as the predictor to train a model. When BioClinicalBERT is applied to the “Note” task, the WordPiece tokenizer [19–21] truncates input text to 512 tokens. This truncation does not occur for other models. For equitable comparison across models, we define the “Note512” task, which truncates the note text to the first 512 tokens used by the BioClinicalBERT model.

### Statistical analysis and modeling

Four model architectures with different conceptual underpinnings were trained: (1) Random forest (RF) [9], (2) Support vector machine (SVM) [11], (3) fastText, [12, 13], and (4) BioClinicalBERT [22]. Each model architecture was trained on each of the 8 prediction tasks for a total of 32 final models.

Each model was trained on the training dataset. Model hyperparameters were tuned using Tune [23] with the BlendSearch [24, 25] algorithm to maximize Matthew’s Correlation Coefficient (MCC) computed on the validation dataset. The number of hyperparameter tuning trials was selected to be 20 times the number of model hyperparameters with early stopping if the MCC of the last 3 trials reaches a plateau with standard deviation < 0.001. The best model was then evaluated on the held-out test dataset. Details on the approach taken for each of the four model architectures is available in Supplemental methods.

### Baseline models

Two baseline models were created for comparison: a random classifier model and an age & medications classifier model. The random classifier model generates a random prediction without using any features, thus serving as a negative control baseline. The age & medications classifier model serves as a simple clinical baseline model. It uses the patient’s age, medication list, and total medication count as input features to a multiclass logistic regression model with cross-entropy loss and L2 penalty for predicting the modified ASA-PS outcome variable. Defaults were used for all other model parameters. Both baselines were implemented using Scikit-learn.

### Evaluation metrics

Final models were evaluated on the held-out test dataset by computing both class-specific and class-aggregate performance metrics. Class-specific metrics include: receiver operator characteristic (ROC) curve, area under receiver operator curve (AUROC), precision-recall curve, area under precision-recall curve (AUPRC), precision (positive predictive value), recall (sensitivity), and F1. Class-aggregate performance metrics include MCC and AUCμ, [26] a multiclass generalization of the binary AUROC. Additionally, macro-average AUROC, AUPRC, precision, recall and F1 were also computed. Each metric and model-task combination was computed with 1000 bootstrap iterations each with 100,000 bootstrap samples on the test set. For each metric, p-values were computed for all 400 pairwise model-task comparisons with the Mann–Whitney U test followed by Benjamini–Hochberg procedure to control false discovery rate with α = 0.01.

### Model interpretability and error analysis

4-by-4 contingency tables were generated to visualize the distribution of model errors. Catastrophic errors were defined as cases where the model predicts ASA IV-V but the anesthesiologist assigned ASA I, or vice versa. For catastrophic errors made by the BioClinicalBERT model with the Note512 task, three new anesthesiologist raters independently assigned an ASA-PS based on only the input text from the Note512 task. These new ASA-PS ratings were compared against the original anesthesiologist’s ASA-PS as well as the model prediction’s ASA-PS.

The SHAP [27] python package was used to train a Shapley values feature attribution model on the test dataset to understand which words support prediction of each modified ASA-PS outcome variable. An analysis of model errors with Shapley value feature attributions was reviewed for each of the catastrophic error examples with representative examples included in the manuscript. Shapley values for predicting each ASA-PS are visualized as a heatmap over text examples. Text examples are de-identified by replacing ages, dates, names, locations, and entities with pseudonyms to achieve data obfuscation while preserving structural similarity to the original passage.

## Results

**Fig. 2:**
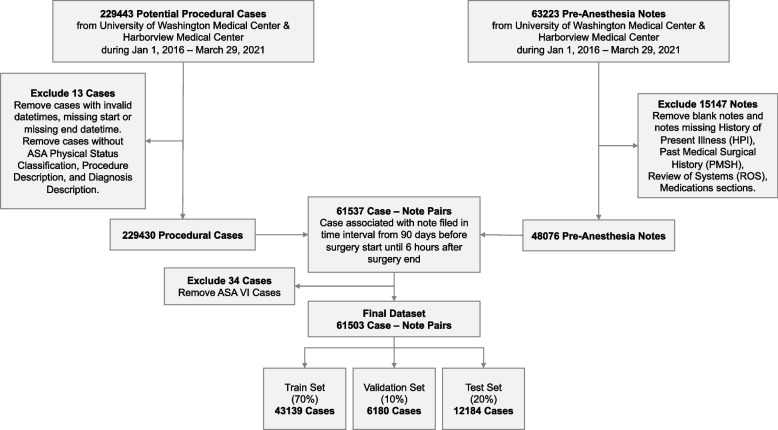
CONSORT Flow Diagram for Dataset Creation. If a patient has multiple procedural cases and pre-anesthesia notes, all of a patient’s cases and notes are allocated to the same data split

Our study comprised 38,566 patients undergoing 61,503 procedures with anesthesia care with 46,275 notes. Baseline patient, procedure, and note characteristics are described in Table 1. A flow diagram describing dataset creation is shown in Fig. 2. A total of 30 class-aggregate and class-specific metrics were computed; 400 pairwise comparisons exist for each metric resulting in 12,000 pairwise comparisons. Only 20 of these pairwise comparisons are not statistically significant (Supplemental Tables 7 and 8). All comparisons across the same model type and varying the task, or across the same task and varying model are statistically significant for reported metrics.

**Table 1:** Dataset characteristics

			**Train**	**Validation**	**Test**
**Patient Characteristics**	**Patient Count, no. (% across dataset splits)**		26994 (70.0%)	3858 (10.0%)	7714 (20.0%)
	**Number of Procedures per Patient, no. (% within dataset split)**	**1**	19107 (70.78%)	2741 (71.05%)	5475 (70.97%)
		**2**	4528 (16.77%)	608 (15.76%)	1330 (17.24%)
		**3**	1635 (6.06%)	249 (6.45%)	425 (5.51%)
		**4**	715 (2.65%)	124 (3.21%)	224 (2.9%)
		**> = 5**	1009 (3.74%)	136 (3.53%)	260 (3.37%)
	**Age, mean (SD)**		50.59 (18.16)	51.51 (18.09)	50.66 (18.0)
	**Gender, no. (% within dataset split)**	**Female**	18419 (42.70%)	2534 (41.00%)	5130 (42.10%)
		**Male**	24720 (57.30%)	3646 (59.00%)	7053 (57.89%)
		**Unknown**	0 (0.0%)	0 (0.0%)	1 (0.01%)
**Procedural Case Characteristics**	**Case Count, no. (% across dataset splits)**		43139 (70.14%)	6180 (10.05%)	12184 (19.81%)
	**Anesthesia Type, no. (% within dataset split)**	**General**	34901 (81.07%)	4961 (80.51%)	9927 (81.64%)
		**MAC**	7063 (16.41%)	1005 (16.31%)	1905 (15.67%)
		**Regional**	1089 (2.53%)	196 (3.18%)	327 (2.69%)
	**ASA Physical Status Classification Score, no. (% within dataset split)**	**I**	3734 (8.66%)	555 (8.98%)	1127 (9.25%)
		**II**	13631 (31.6%)	1875 (30.34%)	3806 (31.24%)
		**III**	18626 (43.18%)	2649 (42.86%)	5327 (43.72%)
		**IV-V**	7148 (16.57%)	1101 (17.82%)	1924 (15.79%)
	**Time Between Pre-Anesthesia Note and Surgery, median days HH:MM:SS (IQR)**		0 days 17:11:48 (0 days 00:17:00, 4 days 06:04:05)	0 days 17:28:55 (0 days 00:18:00, 4 days 05:04:10)	0 days 17:29:55 (0 days 00:17:05, 4 days 01:52:53)
**Note Characteristics**	**Notes Count, no. (% across dataset splits)**		32444 (70.11%)	4649 (10.05%)	9182 (19.84%)
	**Text Word-Level Length, median (IQR)**	**Full Note**	727 (514, 999)	723 (514, 1010)	722 (511, 997)
		**Procedure**	5 (4, 8)	5 (4, 8)	5 (4, 8)
		**Diagnosis**	3 (2, 5)	3 (2, 5)	3 (2, 5)
		**HPI**	86 (35, 162)	87 (35, 161)	88 (35, 163)
		**PMSH**	28 (18, 42)	28 (19, 44)	28 (18, 42)
		**ROS**	87 (53, 154)	87 (54, 155)	87 (54, 153)
		**Medications**	145 (59, 264)	143 (59, 264)	146 (57, 262)

Baseline patient, procedure, and note characteristics for Train, Validation, Test datasets

AUROC for each model architecture and task is shown in Table 2; AUPRC is shown in Table 3; AUCµ and MCC is shown in Supplemental Table 1. RF, SVM, and fastText perform best using the entire note compared to note sections. Tasks with longer text snippets yielded better performance–HPI, ROS and Meds sections result in better model performance as compared to Diagnosis, Procedure, and PMSH. On the Note task, fastText performs the best. On the Note512 task, BioCinicalBERT performs the best.

**Table 2:** Area under receiver operator characteristic for all models

**A. Macro-average AUROC**									
	**Baseline**	**Diagnosis**	**Procedure**	**HPI**	**PMSH**	**ROS**	**Meds**	**Note**	**Note512**
**Random Classifier**	0.500	---	---	---	---	---	---	---	---
**Age & Meds**	0.709	---	---	---	---	---	---	---	---
**Random Forest**	---	0.741	0.751	0.788	0.695	0.778	0.781	0.820	0.802
**Support Vector Machine**	---	0.714	0.717	0.789	0.697	0.787	0.768	0.850	0.829
**fastText**	---	0.757	0.758	0.791	0.720	0.793	0.789	0.865	0.844
**BioClinicalBERT**	---	0.767	0.755	0.814	0.737	0.806	0.784	0.843	0.845
**B. Class-specific AUROC**									
	**Baseline**	**Diagnosis**	**Procedure**	**HPI**	**PMSH**	**ROS**	**Meds**	**Note**	**Note512**
**Random Classifier**									
**I**	0.500	---	---	---	---	---	---	---	---
**II**	0.500	---	---	---	---	---	---	---	---
**III**	0.500	---	---	---	---	---	---	---	---
**IV-V**	0.500	---	---	---	---	---	---	---	---
**Age & Meds**									
**I**	0.863	---	---	---	---	---	---	---	---
**II**	0.638	---	---	---	---	---	---	---	---
**III**	0.668	---	---	---	---	---	---	---	---
**IV-V**	0.668	---	---	---	---	---	---	---	---
**Random Forest**									
**I**	---	0.790	0.810	0.864	0.810	0.869	0.861	0.898	0.886
**II**	---	0.708	0.713	0.744	0.636	0.729	0.738	0.783	0.759
**III**	---	0.660	0.674	0.708	0.644	0.708	0.718	0.747	0.719
**IV-V**	---	0.804	0.806	0.835	0.691	0.803	0.807	0.854	0.844
**Support Vector Machine**									
**I**	---	0.776	0.793	0.874	0.827	0.904	0.869	0.938	0.924
**II**	---	0.653	0.633	0.738	0.592	0.691	0.680	0.806	0.775
**III**	---	0.639	0.650	0.709	0.655	0.728	0.702	0.775	0.750
**IV-V**	---	0.789	0.794	0.836	0.714	0.826	0.821	0.881	0.865
**fastText**									
**I**	---	0.815	0.820	0.870	0.833	0.889	0.863	0.943	0.930
**II**	---	0.724	0.718	0.755	0.675	0.771	0.755	0.833	0.809
**III**	---	0.684	0.685	0.720	0.668	0.729	0.724	0.798	0.771
**IV-V**	---	0.805	0.811	0.819	0.702	0.782	0.815	0.884	0.867
**BioClinicalBERT**									
**I**	---	0.838	0.816	0.901	0.851	0.902	0.861	0.917	0.922
**II**	---	0.711	0.707	0.768	0.674	0.748	0.737	0.806	0.804
**III**	---	0.688	0.681	0.741	0.682	0.752	0.719	0.776	0.779
**IV-V**	---	0.830	0.818	0.848	0.741	0.823	0.818	0.874	0.874

(A) Macro-average AUROC and (B) class-specific AUROC for each model architecture and task on the held-out test set compared to baseline models. Random Classifier serves as a negative control baseline. Age & Meds classifier serves as a simple clinical baseline. Supplemental Table 5 is a copy of this table with all standard errors reported

**Table 3:** Area Under Precision-Recall Curve

**A. Macro-average AUPRC**									
	**Baseline**	**Diagnosis**	**Procedure**	**HPI**	**PMSH**	**ROS**	**Meds**	**Note**	**Note512**
**Random Classifier**	0.250	---	---	---	---	---	---	---	---
**Age & Meds**	0.416	---	---	---	---	---	---	---	---
**Random Forest**	---	0.457	0.462	0.510	0.392	0.484	0.489	0.567	0.534
**Support Vector Machine**	---	0.443	0.451	0.525	0.413	0.514	0.490	0.627	0.593
**fastText**	---	0.478	0.473	0.518	0.421	0.512	0.495	0.642	0.607
**BioClinicalBERT**	---	0.486	0.473	0.570	0.446	0.536	0.499	0.616	0.619
**B. Class-specific AUPRC**									
	**Baseline**	**Diagnosis**	**Procedure**	**HPI**	**PMSH**	**ROS**	**Meds**	**Note**	**Note512**
**Random Classifier**									
**I**	0.091	---	---	---	---	---	---	---	---
**II**	0.316	---	---	---	---	---	---	---	---
**III**	0.429	---	---	---	---	---	---	---	---
**IV-V**	0.163	---	---	---	---	---	---	---	---
**Age & Meds**									
**I**	0.384	---	---	---	---	---	---	---	---
**II**	0.425	---	---	---	---	---	---	---	---
**III**	0.568	---	---	---	---	---	---	---	---
**IV-V**	0.289	---	---	---	---	---	---	---	---
**Random Forest**									
**I**	---	0.285	0.285	0.394	0.295	0.374	0.327	0.488	0.455
**II**	---	0.490	0.487	0.518	0.425	0.515	0.498	0.580	0.550
**III**	---	0.565	0.576	0.614	0.551	0.610	0.621	0.650	0.625
**IV-V**	---	0.488	0.500	0.514	0.299	0.437	0.510	0.550	0.508
**Support Vector Machine**									
**I**	---	0.272	0.305	0.436	0.323	0.433	0.345	0.606	0.575
**II**	---	0.460	0.441	0.519	0.392	0.493	0.477	0.614	0.574
**III**	---	0.568	0.567	0.618	0.570	0.639	0.618	0.684	0.655
**IV-V**	---	0.473	0.492	0.527	0.367	0.491	0.519	0.605	0.568
**fastText**									
**I**	---	0.317	0.308	0.428	0.316	0.429	0.340	0.617	0.575
**II**	---	0.507	0.491	0.531	0.453	0.559	0.517	0.645	0.605
**III**	---	0.590	0.583	0.620	0.568	0.617	0.622	0.705	0.675
**IV-V**	---	0.495	0.510	0.491	0.349	0.444	0.502	0.601	0.575
**BioClinicalBERT**									
**I**	---	0.330	0.301	0.529	0.354	0.445	0.337	0.582	0.591
**II**	---	0.499	0.487	0.562	0.454	0.553	0.521	0.616	0.612
**III**	---	0.599	0.585	0.641	0.588	0.655	0.628	0.679	0.690
**IV-V**	---	0.517	0.519	0.546	0.388	0.492	0.509	0.588	0.585

A) Macro-average AUPRC and (B) class-specific AUPRC for each model architecture and task on the held-out test set compared to baseline models. Random Classifier serves as a negative control baseline. Age & Meds classifier serves as a simple clinical baseline. Supplemental Table 6 is a copy of this table with all standard errors reported

**Fig. 3:**
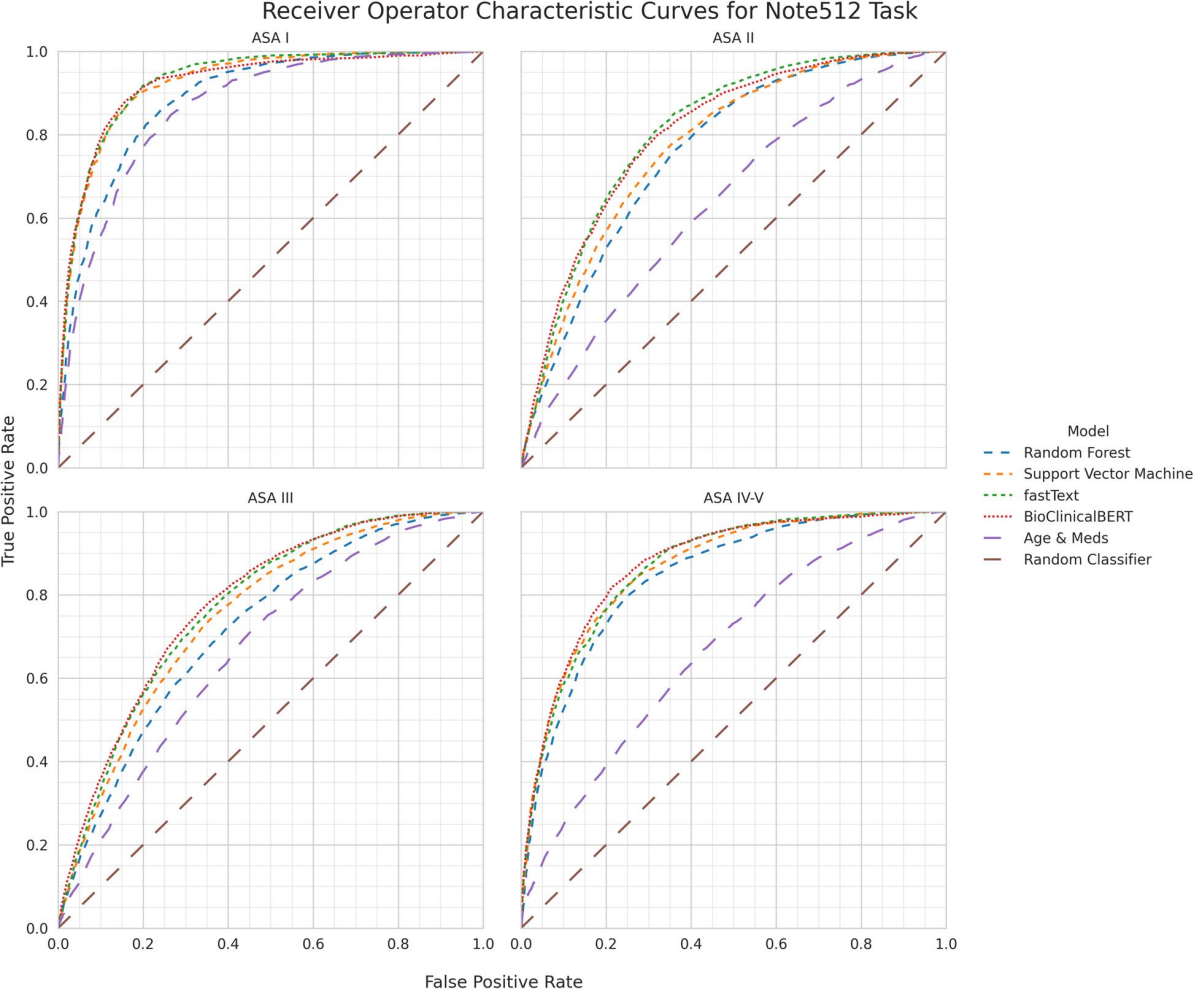
ROC performance of each model architecture on the Note512 task compared to baseline models. Each plot depicts model performance for predicting a specific ASA-PS

**Fig. 4:**
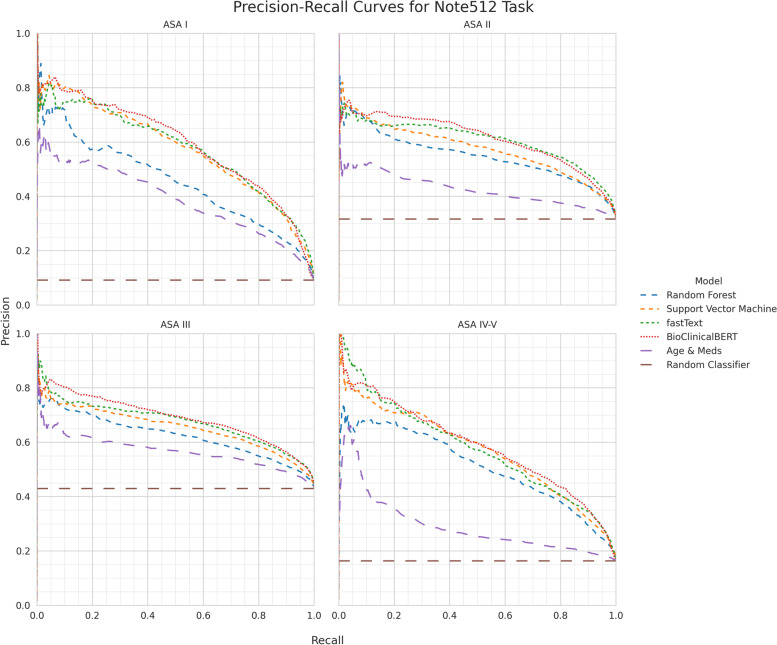
Precision-recall curve performance of each model architecture on the Note512 task compared to baseline models. Each plot depicts model performance for predicting a specific ASA-PS

Direct comparison of models is most appropriate using the Note512 task since all models are given the same information content. For the Note512 task, BioClinicalBERT has better class-aggregate performance across AUROC, AUPRC, AUCμ, MCC, F1 (Supplemental Table 2) compared to other models. While F1 for both fastText and BioClinicalBERT are similar, fastText achieves this with higher macro-precision (positive predictive value) (Supplemental Table 3) whereas BioClinicalBERT achieves this with higher macro-recall (sensitivity) (Supplemental Table 4). Class-specific metrics show that fastText’s worse recall is due to imbalanced recall performance with higher recall for ASA II and III which are the most prevalent classes, but poor recall for ASA I and IV-V. Conversely BioClinicalBERT has worse precision than fastText on all classes except for ASA III. BioClinicalBERT has similar or better AUROC and AUPRC across all the ASA-PS classes. This is also seen in the ROC curves (Fig. 3) and the precision-recall curves (Fig. 4), in which the BioClinicalBERT model shows slightly better performance across most thresholds.

**Fig. 5:**
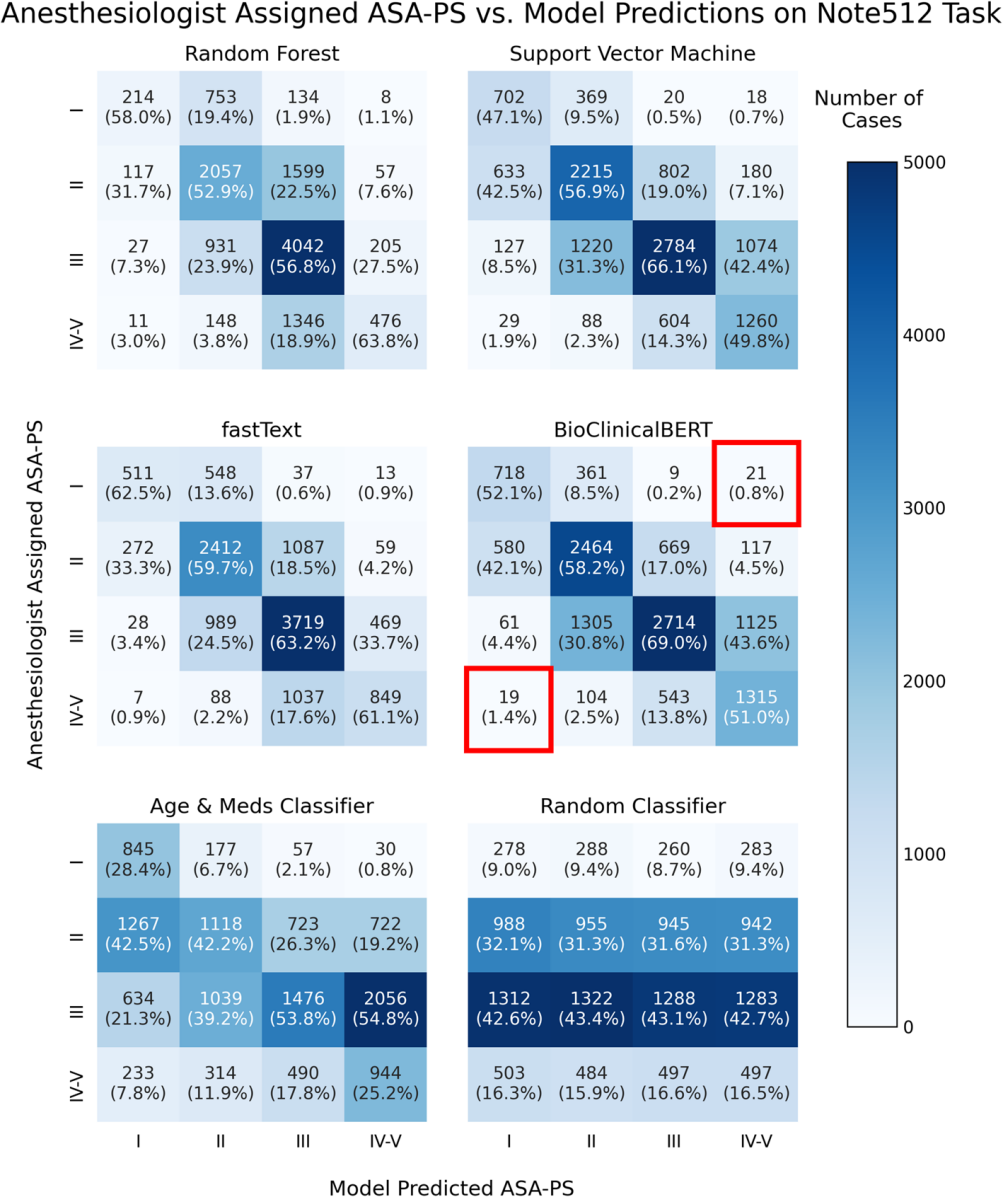
4-by-4 contingency tables for each model architecture on the Note512 task. The vertical axis corresponds to modified ASA-PS recorded in the anesthetic record by the anesthesiologist. The horizontal axis corresponds to the model predicted modified ASA-PS. Numbers in the table represent case count from the test set. Percentages are case counts normalized over the model predicted ASA-PS, representing the distribution of actual ASA-PS recorded in the anesthetic record for a specific model predicted ASA-PS. Cells outlined in red in the BioClinicalBERT contingency table correspond to our definition of catastrophic errors. The 21 cases where anesthesiologist assigned ASA I and BioClinicalBERT model predicted ASA IV-V comprise 1.7% of all cases. The 19 cases where anesthesiologist assigned ASA IV-V and BioClinicalBERT model predicted ASA I comprise 1.6% of all cases

**Fig. 6:**
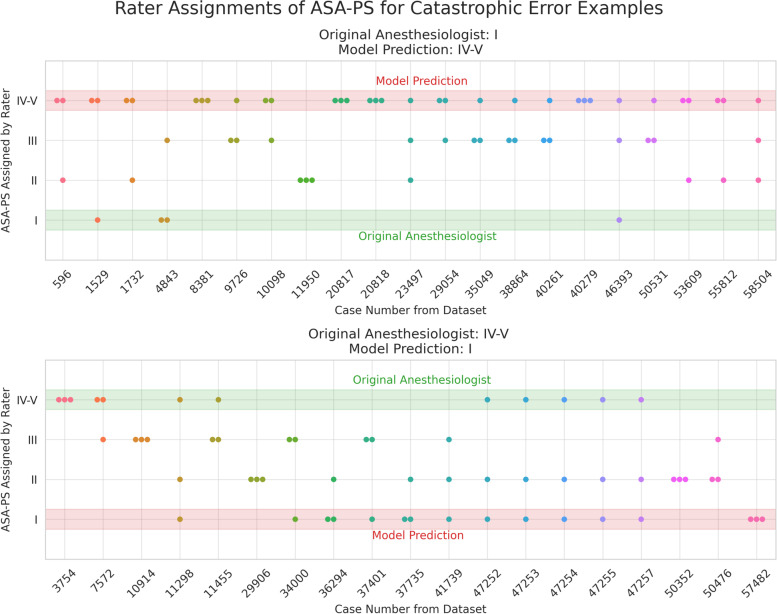
Rater assignments of ASA-PS for catastrophic error examples from the BioClinicalBERT model on Note512 task. Top plot shows scenario where model prediction is ASA IV-V, but original anesthesiologist assigned case ASA I. Bottom plot shows scenario where model prediction is ASA I, but original anesthesiologist assigned case ASA IV-V. Three anesthesiologist raters were asked to read the input text from the Note512 task and assign an ASA-PS for each of the catastrophic error examples. For each case, a dot marks a rater’s ASA-PS assignment. The model’s prediction and original anesthesiologist ASA-PS is shown as a highlighted region overlaid on the plots. Shapley feature attribution visualizations are shown for cases #57482 (Fig. 7, Supplemental Fig. 2), #41739 (Supplemental Fig. 3), #11950 (Supplemental Fig. 4), #29054 (Supplemental Fig. 5)

Figure 5 depicts 4-by-4 contingency tables to visualize distribution of model errors on the Note512 task. When erroneous predictions occur, they are typically adjacent to the ASA-PS assigned by the original anesthesiologist. In the analysis of 40 catastrophic errors made by the BioClinicalBERT model on the Note512 task, the mean absolute difference between the model prediction and a new anesthesiologist rater is 1.025 whereas the difference from the original anesthesiologist is 3 (Fig. 6). This disparity with the original anesthesiologist and greater concordance with the new anesthesiologist rater indicates that some of the “incorrect predictions” on the test set are not true failures of the model but issues with data quality documented in routine clinical care.

**Fig. 7:**
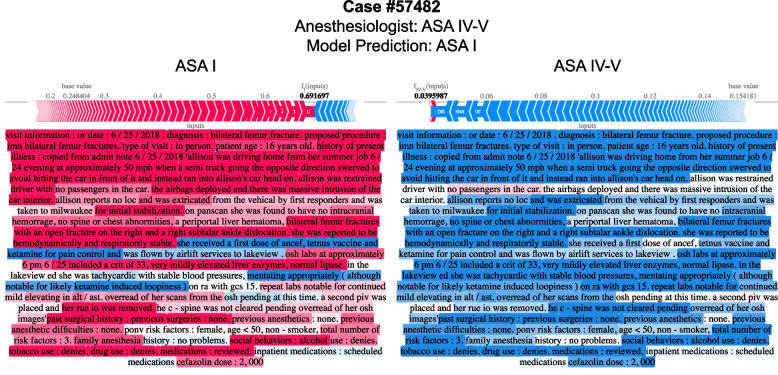
Attribution of input text features to predicting modified ASA-PS for the BioClinicalBERT model on Note512 task. Shapley values for each text token is shown to compare feature attributions to ASA I (top) and feature attributions to ASA IV-V (bottom). Red tokens positively support predicting the target ASA-PS whereas blue tokens do not support predicting the target ASA-PS. The magnitude and direction of support is overlaid on a force plot above the text. The baseline probability of predicting each class in the test set is shown as the “base value” on the force plot. The base value + sum of Shapley values from each token corresponds to the probability of predicting the ASA-PS and is shown as the bolded number. For simplicity, feature attributions to ASA II and III are omitted in this figure, but a full-visualization with all outcome ASA-PS for this text snippet is available in Supplemental Fig. 2. Text examples are de-identified by replacing ages, dates, names, locations, and entities with pseudonyms to achieve data obfuscation while preserving structural similarity to the original passage

Shapley values in Fig. 7 provide clinically plausible explanations for model explanations, highlighting the directional probability of how specific input text contributes to predicting a specific ASA-PS. These feature attributions often provide clinically plausible explanations for why a model is making a wrong prediction and allows the clinician to evaluate the evidence the model is considering. Additional examples shown in Supplemental Figs. 2, 3, 4 and 5.

## Discussion

In this study of ASA-PS prediction using NLP techniques, we found that more advanced models made fewer categorization errors. Further, an assessment of catastrophic errors made by the BioClinicalBERT model suggests that, in the majority of cases, expert review suggested the initial ASA-PS score assigned by the anesthesiologist was erroneous rather than the ASA-PS score assigned by the NLP model. Shapley value feature attributions enable a clinician to easily identify if the model predictions are erroneous or clinically plausible. From these feature attributions, we find NLP models are able to associate both obvious and subtle clinical cues to the patient’s illness severity.

Text classification techniques have undergone substantial evolution over the past decade. Most of these techniques will be unfamiliar to the practicing clinician. In brief, RF and SVM represent more rudimentary approaches that utilize bag-of-words and n-grams. These techniques are sensitive to word misspellings, cannot easily account for word order, have difficulty in capturing long-range references within sentences, and have difficulty in representing different meanings of a word when the same word appears in different contexts [28–33].

Modern NLP techniques have overcome many of these challenges with vector space representation of words [12, 13, 34–36] and subword components [13, 19, 20, 37] as seen in the fastText model, attention mechanism [38, 39], and pretrained deep autoregressive neural networks [40–42] such as transformer neural networks [43]. This has resulted in successful large language models such as BERT [21, 44] and the domain-specific BioClinicalBERT [22]. Perhaps the most widely known large language model is ChatGPT (OpenAI, San Francisco, CA), a general purpose chatbot based on the GPT-3 model which contains 175 billion parameters [45]. In contrast, BioClinicalBERT used in this feasibility study contains roughly 1500 times fewer parameters, but has been trained specifically on clinical notes which makes it well suited for the ASA-PS prediction task [46].

Longer text length provides more information for the model to make an accurate prediction. Even though text snippets such as Diagnosis or Procedure may have high relevance for the illness severity of the patient, the better performance on longer input text sequences indicate that more information is generally better. This is similar to what is observed in the multifaceted practice of clinical medicine–where a patient’s overall clinical status is often better understood as the sum of many weaker but synergistic signals rather than a single descriptor. The limited input sequence length for BioClinicalBERT creates a performance ceiling as it limits the amount of information available to the model. Comparing Note and Note512 tasks, all other models that can utilize the full note have better performance when this input length is lifted with fastText being the top performer. These findings suggest that future development of a large language model similar to BioClinicalBERT capable of accepting a longer input context would likely have superior performance characteristics. fastText requires significantly less compute resources for model training and inference compared to BioClinicalBERT and remains a good option in lower resource settings. RF and SVM were our worst performing models, confirming that modern word vector and neural network language model-based approaches are superior.

There is significant variability on the length and quality of clinical free-form text narrative written in the note, especially in the HPI section which is typically a clinician’s narrative of the patient’s medical status and need for the procedure. In some cases, the HPI section contains one or two words in length (Supplemental Fig. 4), whereas in other cases it is a rich narrative (Supplemental Figs. 2, 5). We believe that relatively poor performance in the ASA-PS prediction using HPI alone is a consequence of variability in documentation, as the model may have limited information for prediction if the note text does not richly capture the clinical scenario.

These models rarely made catastrophic errors. Erroneous predictions are typically adjacent to the ASA-PS assigned by the anesthesiologist, suggesting the model is making appropriate associations between freeform text predictors and the outcome variable (Fig. 5). Furthermore, when new anesthesiologist raters were asked to assign ASA-PS to the cases where catastrophic errors occurred from the BioclinicalBERT model on the Note512 task, there was greater concordance between the model predictions and the new anesthesiologist rather than the original anesthesiologist (Fig. 6). Shapley feature attributions for one of these catastrophic errors in Fig. 7 reveal that the original anesthesiologist may have made the wrong assignment, or may have written a note that does not reflect the true clinical scenario. In this example, the original anesthesiologist assigned the case ASA IV-V, but the model predicted I. Feature attributions show the BioClinicalBERT model correctly identifies pertinent negatives on trauma exam, normal hematocrit of 33, and normal Glasgow Coma Scale (GCS) of 15 to all support a prediction for ASA I and against ASA IV-V [47]. In this example, all new anesthesiologist raters agree with the model rather than the original anesthesiologist. These findings from our catastrophic error analysis suggest that the model performance may be underestimated by our evaluation metrics, as our ground truth test set contains imperfect ASA-PS assignments. It also illustrates how the model is robust against potentially faulty labels. Despite a noisy training and evaluation set, NLP models are still able to make clinically appropriate ASA-PS predictions.

Our exploration of Shapley feature attributions reveal that the model is able to identify indirect indicators of a patient’s illness severity. For example, subcutaneous heparin is often administered for bed-bound inpatients to prevent the development of deep vein thrombosis. Supplemental Fig. 4 depicts an example where the model learns to associate mention of subcutaneous heparin in the medication list with a higher ASA-PS, likely because hospitalized patients are generally more ill than outpatients who present to the hospital for same-day surgery. Similarly, the model learns the association between the broad spectrum antibiotic ertapenem with a higher ASA-PS as compared to narrow spectrum or prophylactic antibiotics such as metronidazole or cefazolin. These observations show that the model is able to identify and link these subtle indicators to a patient’s illness severity. Shapley value feature attributions prove to be an effective tool that enables clinicians to understand how a model makes its prediction from text predictors.

## Limitations

Our dataset is derived from a real-world EHR used to provide clinical care and includes human and computer generated errors. These issues include data entry and spelling, the use of abbreviations, references to other notes and test results not available to the model, and automatically generated/inserted text as part of a note template. For this feasibility study we use the anesthesia preoperative evaluation note. This note is typically written days or weeks in advance for elective procedures, but is sometimes written immediately prior, during, or after the procedure in urgent or emergent scenarios. These notes are included because our goal is to study the factors that affect ASA-PS prediction using note text with NLP models. We have not conducted clinical validation of these models and we have not validated model performance across multiple institutions.

The BioClinicalBERT model is limited to an input sequence of 512 tokens; future investigation is needed to understand if longer-context large language models can achieve better performance. We also did not explore more advanced NLP models such as those that perform entity and relation extraction, which may further enhance the prediction performance. Larger model sizes such as GPT-3 have been shown to be correlated with improved model performance across a variety of tasks, but these models are not specialized for the clinical domain; we do not explore these models in our feasibility study and leave this exploration to future research [48].

Finally, the ASA-PS is known to have only moderate interrater agreement among human anesthesiologists [49, 50]. Consequently, a perfect classification on this task is not possible since the ground truth labels derived from the EHR encapsulate this interrater variability.

## Conclusions

Our feasibility assessment suggests that NLP models can accurately predict a patient’s illness severity using only free-form text descriptions of patients without any manual data extraction. They can be automatically applied to entire panels of patients, potentially allowing partial automation of preoperative assessment triage while also serving as a measure of perioperative risk stratification. Clinical decision support tools could use techniques like these to improve identification of comorbidities, resulting in improved patient safety. These tools may also be used at the healthcare system level for population health analyses and for billing purposes. Predictions made by more advanced NLP models benefit from explainability through Shapley feature attributions, which produce explanations that logically support model predictions and are understandable to clinicians. Future work includes assessment of more advanced natural language models that have more recently become available, use of non-anesthesiologist clinician notes, and exploration of NLP-based prediction of other outcome variables which may be less subject to interrater variability.

### Supplementary Information

**Additional file 1: Supplemental Methods.** Details on the approach taken for each of the four model architectures [51–59].

**Additional file 2: Supplemental Figure 1.** BioClinicalBERT Model Architecture with additional prediction heads for fine-tuning and prediction of modified ASA-PS. **Supplemental Figure 2.** Attribution of input text features to predicting modified ASA-PS for the BioClinicalBERT model on Note512 task. Model prediction is ASA I, Anesthesiologist assigned case ASA IV-V. Notable findings include the model focusing on pertinent negatives on trauma exam and imaging findings and a normal hematocrit of 33 all of which support predicting a ASA-PS I. The same pertinent negatives as well as a Glasgow Coma Scale (GCS) of 15 are negatively Shapley values for ASA-PS IV-V, which reduce the probability of predicting ASA IV-V. Despite the anesthesiologist’s assignment of ASA IV-V, the text description does not suggest the patient has severe systemic disease with constant threat to life (ASA IV) or is moribund and requires the operation to survive (ASA V). Text examples are de-identified by replacing ages, dates, names, locations, and entities with pseudonyms to achieve data obfuscation while preserving structural similarity to the original passage. **Supplemental Figure 3.** Attribution of input text features to predicting modified ASA-PS for the BioClinicalBERT model on Note512 task. Model prediction is ASA I, Anesthesiologist assigned case ASA IV-V. Notable findings include the model associating chest tube with ASA IV-V. The model has trouble with consistently attributing the multiple mentions of eyelid laceration with a specific ASA-PS. The model may be inappropriately assigning mention of left pneumothorax to ASAI. This example depicts a challenge for the model in which a relatively minor injury (eyelid laceration) is simultaneously present with a potentially severe injury (pneumothorax), though the severity of the pneumothorax is not mentioned and thus the text predominantly supports ASA I (healthy) or ASA II (mild systemic disease). This kind of mixed illness/injury example coupled with a narrative that does not clearly describe disease severity may be a struggle for the model. Text examples are de-identified by replacing ages, dates, names, locations, and entities with pseudonyms to achieve data obfuscation while preserving structural similarity to the original passage. **Supplemental Figure 4.** Attribution of input text features to predicting modified ASA-PS for the BioClinicalBERT model on Note512 task. Model prediction is ASA IV-V, Anesthesiologist assigned case ASA I. Notable findings include: young age associated with ASA I and ASA IV-V, but negatively associated with ASA II and III; diagnosis of perforated appendix and procedure of laparoscopic appendectomy negatively associated with ASA I and positively associated with higher ASA-PS; model identifying broad-spectrum antibiotics such as ertapenem to be associated with ASA IV-V, but narrower-spectrum antibiotics such as metronidazole, cefazolin to be heavily associated with ASA I; inpatient medications such as subcutaneous heparin and ondansetron negatively associated with lower ASA-PS and positively associated with higher ASA-PS. Text examples are de identified by replacing ages, dates, names, locations, and entities with pseudonyms to achieve data obfuscation while preserving structural similarity to the original passage. **Supplemental Figure 5.** Attribution of input text features to predicting modified ASA-PS for the BioClinicalBERT model on Note512 task. Model prediction is ASA IV-V, Anesthesiologist assigned case ASA I. Notable findings include medical conditions and interventions associated with higher ASA-PS such as cardiomyopathy, internal cardiac defibrillator (ICD) generator change, paroxysmal ventricular tachycardia, left ventricular assist device (LVAD), heart failure, possible transplantation, tricuspid valve repair, and patent foramen ovale (PFO) closure; history of chronic cigarette smoking and snoring associated with ASA IV-V. The text description is at least ASA III (severe systemic illness), and can be argued to be ASA IV (severe systemic disease with constant threat to life) if heart failure is progressively worsening. In this example the model appears to make a more appropriate ASA-PS prediction than the anesthesiologist. Text examples are de-identified by replacing ages, dates, names, locations, and entities with pseudonyms to achieve data obfuscation while preserving structural similarity to the original passage. **Supplemental Table 1.** (A) Matthew's correlation coefficient (MCC) and (B) AUCµ for each model architecture and task on the held-out test set compared to baseline models. MCC is a categorical analog of Pearson’s correlation coefficient. AUCµ is a multiclass generalization of AUROC and U statistic and is more theoretically grounded than macro-average AUROC, but less commonly reported. Standard errors are reported in parenthesis. **Supplemental Table 2.** (A) Macro-average F1 and (B) class specific F1 for each model architecture and task on the held-out test set compared to baseline models. Standard errors are reported in parenthesis. **Supplemental Table 3.** (A) Macro-average precision and (B) class-specific precision for each model architecture and task on the held-out test set compared to baseline models. Standard errors are reported in parenthesis. **Supplemental Table 4.** (A) Macro-average recall and (B) class-specific recall for each model architecture and task on the held-out test set compared to baseline models. Standard errors are reported in parenthesis. **Supplemental Table 5.** (A) Macro-average AUROC and (B) class-specific AUROC for each model architecture and task on the held-out test set compared to baseline models. Standard errors are reported in parenthesis. **Supplemental Table 6.** (A) Macro-average AUPRC and (B) class-specific AUPRC for each model architecture and task on the held-out test set compared to baseline models. Standard errors are reported in parenthesis. **Supplemental Table 7.** *P*-values for all pairwise comparisons which were not statistically significant. Reported *p*-values are  corrected for multiple hypothesis testing using the Benjamini-Hochberg procedure with α =0.01.

**Additional file 3: Supplemental Table 8.** Statistically Significant Pairwise Metric Comparisons.

## Data Availability

The datasets generated and/or analyzed during the current study are not publicly available because the text dataset derived from electronic health records comprises personal identifiable information (PII) and protected health information (PHI). Data may be requested by contacting Vikas O’Reilly-Shah at voreill@uw.edu or the University of Washington Center for Perioperative & Pain Initiatives in Quality Safety Outcome (PPiQSO) at PPiQSO@uw.edu. Data access is contingent upon a data use agreement in accordance with UW Medicine policy. Code for experiments and results is publicly available at https://github.com/philipchung/nlp-asa-prediction.

## Abbreviations

NLP: Natural Language Processing
EHR: Electronic Health Record
ASA-PS: American Society of Anesthesiologists Physical Status
TFIDF: Term Frequency Inverse Document Frequency
TRIPOD: Transparent Reporting of a Multivariable Prediction Model for Individual Prognosis or Diagnosis
HPI: History of Present Illness
PMSH: Past Medical Surgical History
ROS: Review of Systems
Meds: Medications
Note512: Note truncated to 512 tokens by the BERT WordPiece tokenizer
RF: Random Forest
SVM: Support Vector Machine
MCC: Matthew’s Correlation Coefficient
AUROC: Area Under Receiver Operating Characteristic
AUPRC: Area Under Precision-Recall Curve
AUCμ: Area Under Curve Multiclass U-statistic
F1: Harmonic Mean of Precision and Recall
GPT: Generative Pre-trained Transformer
GCS: Glasgow Coma Scale
