# Supplementary material for: Prediction of American Society of Anesthesiologists Physical Status Classification from preoperative clinical text narratives using natural language processing
Source: BMC Anesthesiol. 2023 Sep 4;23:296. doi: 10.1186/s12871-023-02248-0 (PMC10476287; doi:10.1186/s12871-023-02248-0)
Supplement: Supplementary file 1 — Additional file 1: Supplemental Methods. Details on the approach taken for each of the four model architectures [51–59]. [file 12871_2023_2248_MOESM1_ESM.docx]

## Supplemental Methods

Details on the approach taken for each of the four model architectures.

#### Random Forest

Text input was preprocessed into a unigram and bigram count matrix followed by TFIDF transform without removal of stop words or lemmatization.[[10]](https://paperpile.com/c/4yn9PZ/OnEmk) Random forest classifier from the Scikit-learn[[51]](https://paperpile.com/c/4yn9PZ/gHmJ2) python library was used with minimization of gini impurity objective function and weighting each outcome class by inverse frequency to adjust for class imbalance. Hyperparameters tuned include: number of trees and number of features when looking for best split. Defaults were used for all other model parameters.

#### Support Vector Machine

Text input was preprocessed into a unigram and bigram count matrix followed by TFIDF transform without removal of stop words or lemmatization.[[10]](https://paperpile.com/c/4yn9PZ/OnEmk) LinearSVC[[52]](https://paperpile.com/c/4yn9PZ/AGpd3) from the Scikit-learn[[51]](https://paperpile.com/c/4yn9PZ/gHmJ2) python library was used with minimization of squared hinge loss with L2 penalty[[53]](https://paperpile.com/c/4yn9PZ/Gb4BE) and weighting each outcome class by inverse frequency to adjust for class imbalance. Crammer-Singer approach was used for the multiclass strategy.[[54]](https://paperpile.com/c/4yn9PZ/rqtLh) The “C” regularization strength parameter was tuned as a hyperparameter. Defaults were used for all other model parameters.

#### fastText

Text was directly input into the fastText classification model, which internally combines word and sub-word vector representations using continuous bag-of-words[[34]](https://paperpile.com/c/4yn9PZ/UPDpm) and softmax with negative sampling loss[[35]](https://paperpile.com/c/4yn9PZ/hP7af) objective function. Hyperparameters tuned include: learning rate, learning rate update rate, word vector dimension size, context window size, number of negatives sampled, number of epochs. Defaults were used for all other model parameters.

#### BioClinicalBERT

Text was tokenized using WordPiece tokenizer[[19–21]](https://paperpile.com/c/4yn9PZ/wg3gT+h9blN+qHFsu) and then used as input to a pre-trained BioClinicalBERT model[[22]](https://paperpile.com/c/4yn9PZ/STVRH) with the addition of ASA-PS and Emergency modifier prediction heads, each consisting of a linear and softmax layer, for our specific ASA-PS prediction task (Supplemental Figure 1). These prediction heads were jointly optimized with AdamW optimizer[[55]](https://paperpile.com/c/4yn9PZ/HaknD) during training using a weighted average of the cross-entropy loss from each prediction head; the weight of ASA-PS was held constant at 1.0 and the weight of the emergency modifier head was tuned as a hyperparameter. Cross-entropy loss is weighed by inverse class frequency to adjust for class imbalance. Both tokenizer and model are based on the Hugging Face[[56]](https://paperpile.com/c/4yn9PZ/yDHpZ) python implementation with GPU acceleration enabled by PyTorch[[57]](https://paperpile.com/c/4yn9PZ/xAntk) and PyTorch Lightning[[58]](https://paperpile.com/c/4yn9PZ/8tVV3). Tokenizer and model sequence length were set to the maximum of 512 tokens for the pretrained model. Longer input text sequences were truncated to this length. Hyperparameters tuned include: emergency head weight, batch size, learning rate, weight decay, dropout, gradient clipping, and number of epochs. ASHA[[59]](https://paperpile.com/c/4yn9PZ/qFDGu) with a reduction factor of 3 was used to tune up to 4 instances of the same model with different hyperparameters in parallel.
