# Supplementary material for: Prediction of American Society of Anesthesiologists Physical Status Classification from preoperative clinical text narratives using natural language processing
Source: BMC Anesthesiol. 2023 Sep 4;23:296. doi: 10.1186/s12871-023-02248-0 (PMC10476287; doi:10.1186/s12871-023-02248-0)
Supplement: Supplementary file 2 — Additional file 2: Supplemental Figure 1. BioClinicalBERT Model Architecture with additional prediction heads for fine-tuning and prediction of modified ASA-PS. Supplemental Figure 2. Attribution of input text features to predicting modified ASA-PS for the BioClinicalBERT model on Note512 task. Model prediction is ASA I, Anesthesiologist assigned case ASA IV-V. Notable findings include the model focusing on pertinent negatives on trauma exam and imaging findings and a normal hematocrit of 33 all of which support predicting a ASA-PS I. The same pertinent negatives as well as a Glasgow Coma Scale (GCS) of 15 are negatively Shapley values for ASA-PS IV-V, which reduce the probability of predicting ASA IV-V. Despite the anesthesiologist’s assignment of ASA IV-V, the text description does not suggest the patient has severe systemic disease with constant threat to life (ASA IV) or is moribund and requires the operation to survive (ASA V). Text examples are de-identified by replacing ages, dates, names, locations, and entities with pseudonyms to achieve data obfuscation while preserving structural similarity to the original passage. Supplemental Figure 3. Attribution of input text features to predicting modified ASA-PS for the BioClinicalBERT model on Note512 task. Model prediction is ASA I, Anesthesiologist assigned case ASA IV-V. Notable findings include the model associating chest tube with ASA IV-V. The model has trouble with consistently attributing the multiple mentions of eyelid laceration with a specific ASA-PS. The model may be inappropriately assigning mention of left pneumothorax to ASAI. This example depicts a challenge for the model in which a relatively minor injury (eyelid laceration) is simultaneously present with a potentially severe injury (pneumothorax), though the severity of the pneumothorax is not mentioned and thus the text predominantly supports ASA I (healthy) or ASA II (mild systemic disease). This kind of mixed illness/injury example coupled with [file 12871_2023_2248_MOESM2_ESM.docx]

## Supplemental Figures

### Supplemental Figure 1: BioClinicalBERT Model Used for Fine-Tuning


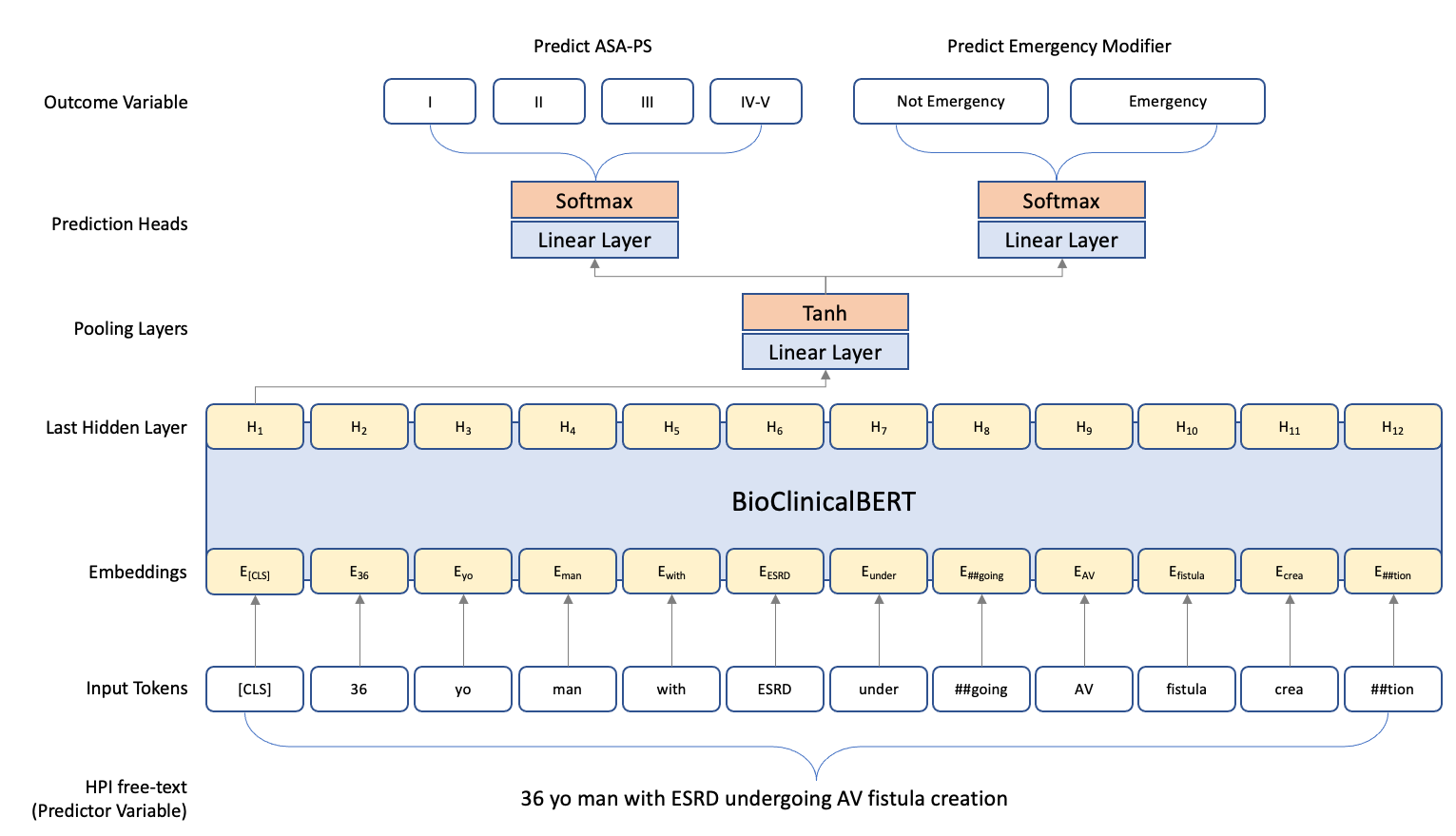


Supplemental Figure 1: BioClinicalBERT Model Architecture with additional prediction heads for fine-tuning and prediction of modified ASA-PS

### Supplemental Figure 2: Shapley Values Overlaid on Note. Example 1.


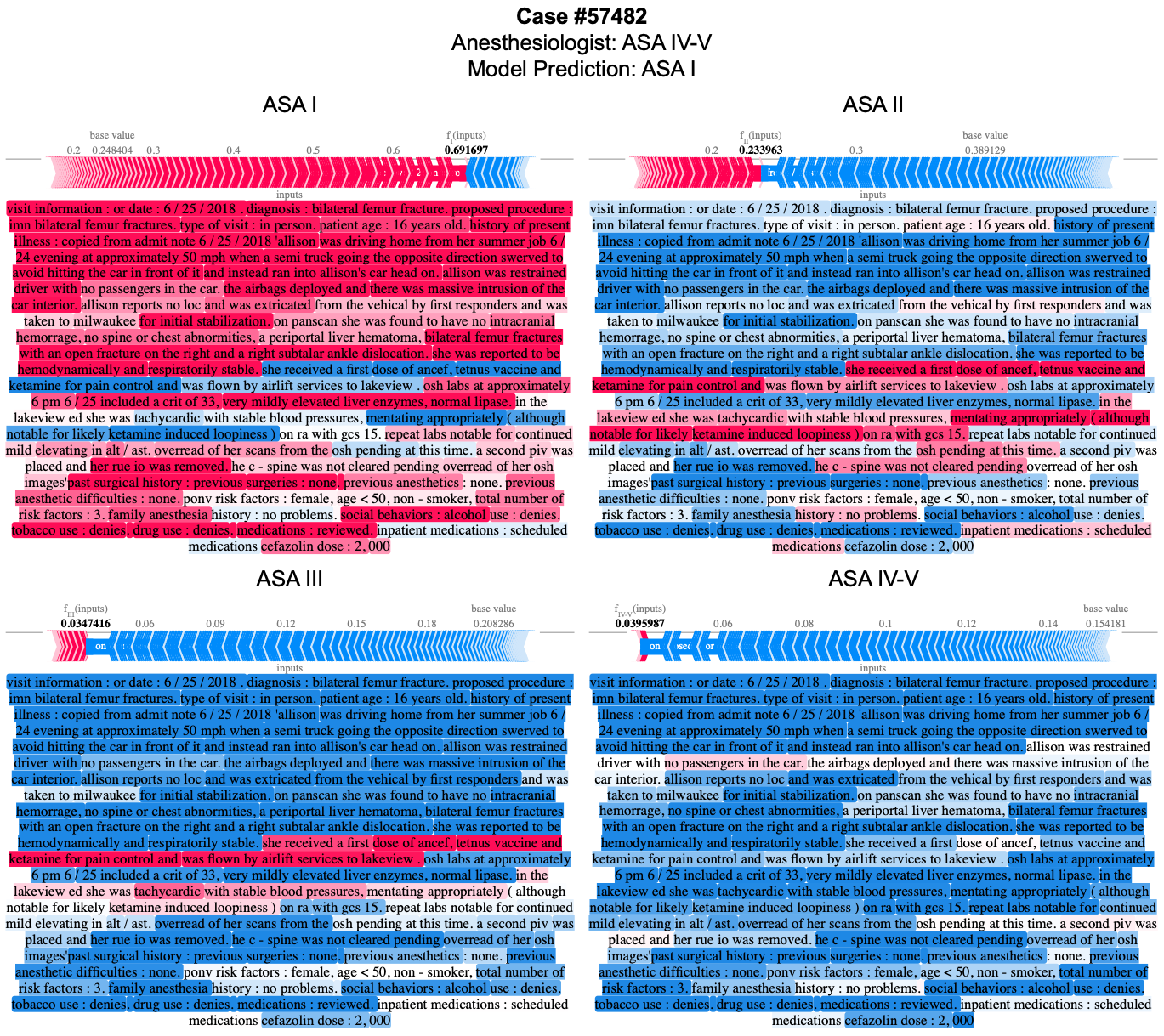


Supplemental Figure 2: Attribution of input text features to predicting modified ASA-PS for the BioClinicalBERT model on Note512 task. Model prediction is ASA I, Anesthesiologist assigned case ASA IV-V. Notable findings include the model focusing on pertinent negatives on trauma exam and imaging findings and a normal hematocrit of 33 all of which support predicting a ASA-PS I. The same pertinent negatives as well as a Glasgow Coma Scale (GCS) of 15 are negatively Shapley values for ASA-PS IV-V, which reduce the probability of predicting ASA IV-V. Despite the anesthesiologist’s assignment of ASA IV-V, the text description does not suggest the patient has severe systemic disease with constant threat to life (ASA IV) or is moribund and requires the operation to survive (ASA V). Text examples are de-identified by replacing ages, dates, names, locations, and entities with pseudonyms to achieve data obfuscation while preserving structural similarity to the original passage.

### Supplemental Figure 3: Shapley Values Overlaid on Note. Example 2.


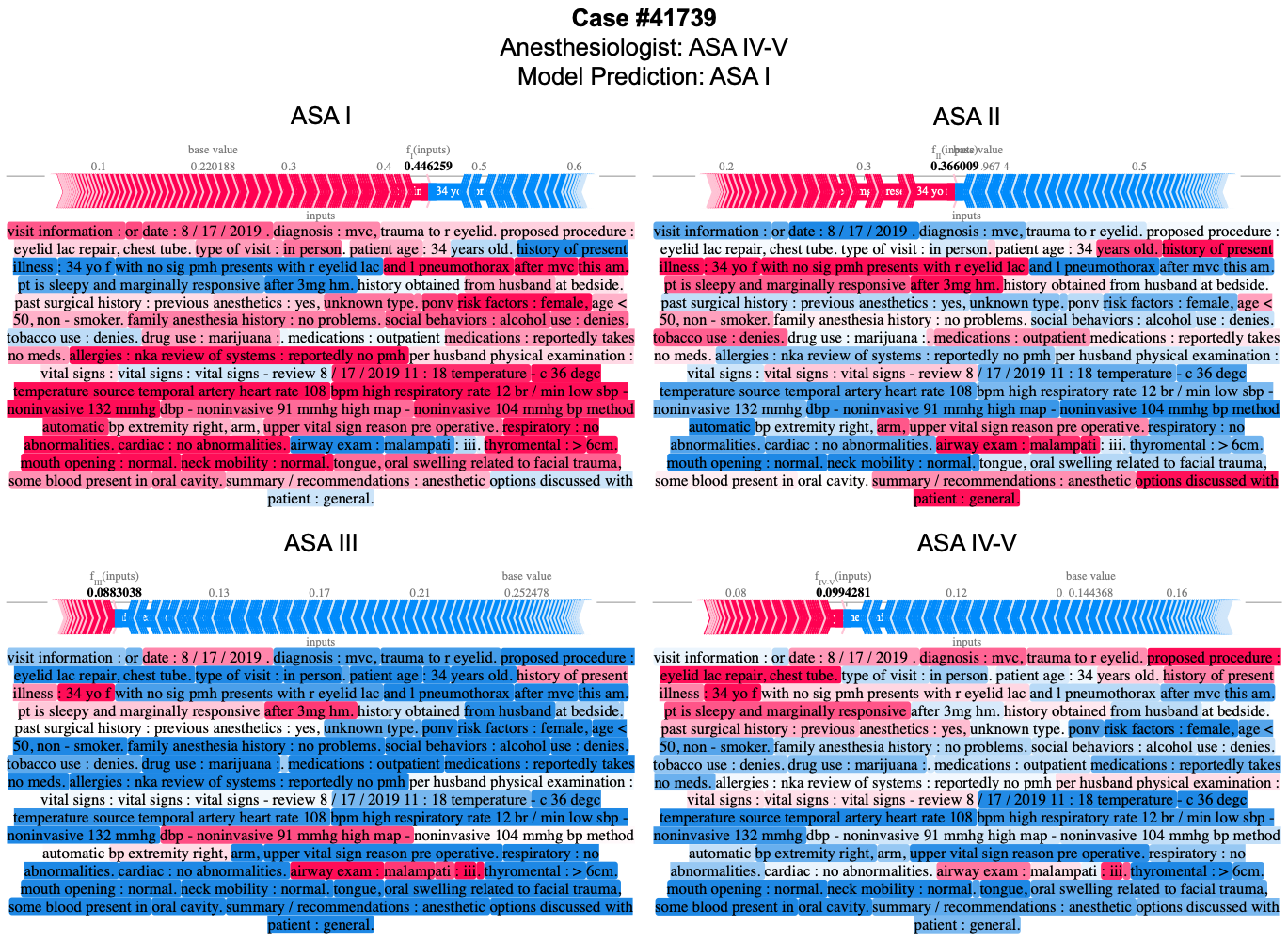


Supplemental Figure 3: Attribution of input text features to predicting modified ASA-PS for the BioClinicalBERT model on Note512 task. Model prediction is ASA I, Anesthesiologist assigned case ASA IV-V. Notable findings include the model associating chest tube with ASA IV-V. The model has trouble with consistently attributing the multiple mentions of eyelid laceration with a specific ASA-PS. The model may be inappropriately assigning mention of left pneumothorax to ASAI. This example depicts a challenge for the model in which a relatively minor injury (eyelid laceration) is simultaneously present with a potentially severe injury (pneumothorax), though the severity of the pneumothorax is not mentioned and thus the text predominantly supports ASA I (healthy) or ASA II (mild systemic disease). This kind of mixed illness/injury example coupled with a narrative that does not clearly describe disease severity may be a struggle for the model. Text examples are de-identified by replacing ages, dates, names, locations, and entities with pseudonyms to achieve data obfuscation while preserving structural similarity to the original passage.

### Supplemental Figure 4: Shapley Values Overlaid on Note. Example 3.


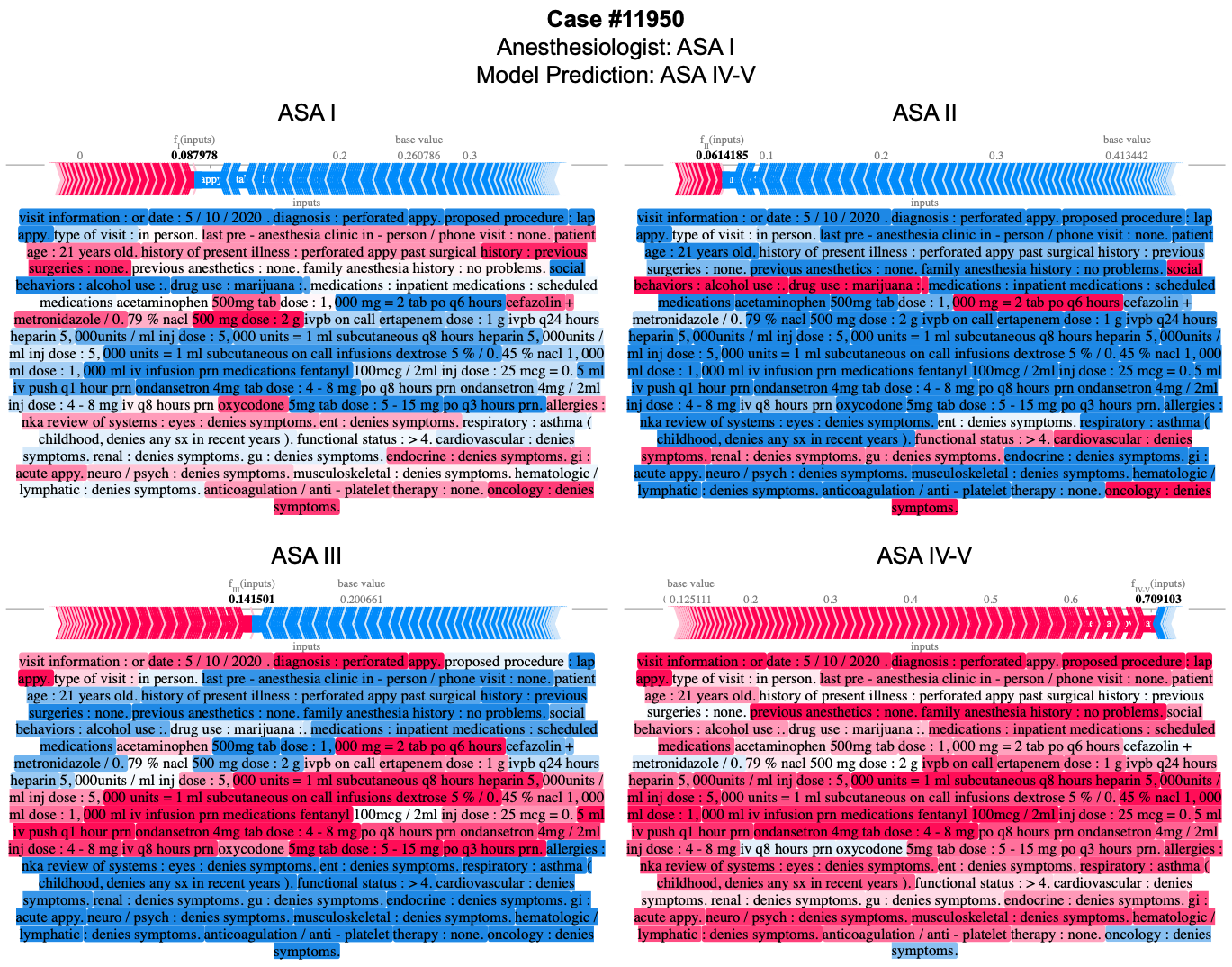


Supplemental Figure 4: Attribution of input text features to predicting modified ASA-PS for the BioClinicalBERT model on Note512 task. Model prediction is ASA IV-V, Anesthesiologist assigned case ASA I. Notable findings include: young age associated with ASA I and ASA IV-V, but negatively associated with ASA II and III; diagnosis of perforated appendix and procedure of laparoscopic appendectomy negatively associated with ASA I and positively associated with higher ASA-PS; model identifying broad-spectrum antibiotics such as ertapenem to be associated with ASA IV-V, but narrower-spectrum antibiotics such as metronidazole, cefazolin to be heavily associated with ASA I; inpatient medications such as subcutaneous heparin and ondansetron negatively associated with lower ASA-PS and positively associated with higher ASA-PS. Text examples are de-identified by replacing ages, dates, names, locations, and entities with pseudonyms to achieve data obfuscation while preserving structural similarity to the original passage.

### Supplemental Figure 5: Shapley Values Overlaid on Note. Example 4.


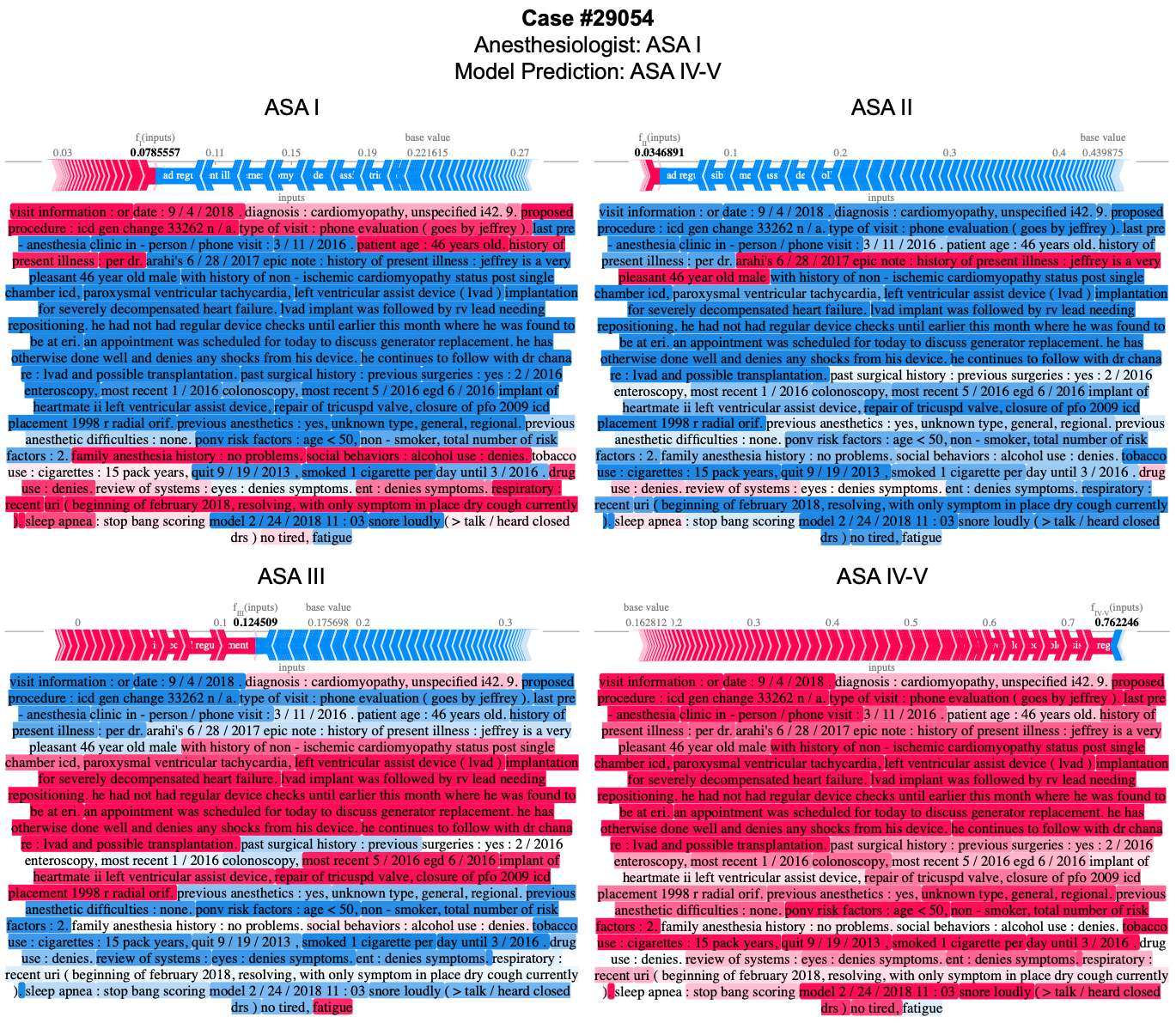


Supplemental Figure 5: Attribution of input text features to predicting modified ASA-PS for the BioClinicalBERT model on Note512 task. Model prediction is ASA IV-V, Anesthesiologist assigned case ASA I. Notable findings include medical conditions and interventions associated with higher ASA-PS such as cardiomyopathy, internal cardiac defibrillator (ICD) generator change, paroxysmal ventricular tachycardia, left ventricular assist device (LVAD), heart failure, possible transplantation, tricuspid valve repair, and patent foramen ovale (PFO) closure; history of chronic cigarette smoking and snoring associated with ASA IV-V. The text description is at least ASA III (severe systemic illness), and can be argued to be ASA IV (severe systemic disease with constant threat to life) if heart failure is progressively worsening. In this example the model appears to make a more appropriate ASA-PS prediction than the anesthesiologist. Text examples are de-identified by replacing ages, dates, names, locations, and entities with pseudonyms to achieve data obfuscation while preserving structural similarity to the original passage.

### Supplemental Table 1: Matthew’s Correlation Coefficient and AUCµ

| **A. Matthew's Correlation Coefficient (MCC)** | | | | | | | | | |
| --- | --- | --- | --- | --- | --- | --- | --- | --- | --- |
|  | **Baseline** | **Diagnosis** | **Procedure** | **HPI** | **PMSH** | **ROS** | **Meds** | **Note** | **Note512** |
| **Random Classifier** | -0.000 (2E-03) | --- | --- | --- | --- | --- | --- | --- | --- |
| **Age & Meds** | 0.172 (2E-03) | --- | --- | --- | --- | --- | --- | --- | --- |
| **Random Forest** | --- | 0.264 (2E-03) | 0.265 (2E-03) | 0.280 (2E-03) | 0.197 (2E-03) | 0.293 (2E-03) | 0.314 (2E-03) | 0.370 (2E-03) | 0.317 (2E-03) |
| **Support Vector Machine** | --- | 0.248 (2E-03) | 0.232 (2E-03) | 0.333 (2E-03) | 0.194 (2E-03) | 0.326 (2E-03) | 0.299 (2E-03) | 0.431 (2E-03) | 0.398 (2E-03) |
| **fastText** | --- | 0.280 (2E-03) | 0.278 (2E-03) | 0.336 (2E-03) | 0.230 (2E-03) | 0.360 (2E-03) | 0.324 (2E-03) | 0.461 (2E-03) | 0.425 (2E-03) |
| **BioClinicalBERT** | --- | 0.280 (2E-03) | 0.267 (2E-03) | 0.370 (2E-03) | 0.226 (2E-03) | 0.364 (2E-03) | 0.321 (2E-03) | 0.439 (2E-03) | 0.430 (2E-03) |

| **B. AUCµ** | | | | | | | | | |
| --- | --- | --- | --- | --- | --- | --- | --- | --- | --- |
|  | **Baseline** | **Diagnosis** | **Procedure** | **HPI** | **PMSH** | **ROS** | **Meds** | **Note** | **Note512** |
| **Random Classifier** | 0.500 (0E+00) | --- | --- | --- | --- | --- | --- | --- | --- |
| **Age & Meds** | 0.768 (1E-03) | --- | --- | --- | --- | --- | --- | --- | --- |
| **Random Forest** | --- | 0.772 (1E-03) | 0.781 (1E-03) | 0.821 (1E-03) | 0.727 (1E-03) | 0.809 (1E-03) | 0.806 (1E-03) | 0.852 (1E-03) | 0.836 (1E-03) |
| **Support Vector Machine** | --- | 0.766 (1E-03) | 0.778 (1E-03) | 0.830 (1E-03) | 0.755 (1E-03) | 0.849 (9E-04) | 0.827 (1E-03) | 0.889 (8E-04) | 0.872 (8E-04) |
| **fastText** | --- | 0.777 (1E-03) | 0.776 (1E-03) | 0.812 (1E-03) | 0.745 (1E-03) | 0.825 (1E-03) | 0.809 (1E-03) | 0.882 (8E-04) | 0.865 (9E-04) |
| **BioClinicalBERT** | --- | 0.817 (1E-03) | 0.792 (1E-03) | 0.850 (1E-03) | 0.791 (1E-03) | 0.846 (9E-04) | 0.814 (1E-03) | 0.869 (9E-04) | 0.876 (9E-04) |

Supplemental Table 1: (A) Matthew's correlation coefficient (MCC) and (B) AUCµ for each model architecture and task on the held-out test set compared to baseline models. MCC is a categorical analog of Pearson’s correlation coefficient. AUCµ is a multiclass generalization of AUROC and U statistic and is more theoretically grounded than macro-average AUROC, but less commonly reported. Standard errors are reported in parenthesis.

###

### Supplemental Table 2: F1 Scores

| **A. Macro-average F1** | | | | | | | | | |
| --- | --- | --- | --- | --- | --- | --- | --- | --- | --- |
|  | **Baseline** | **Diagnosis** | **Procedure** | **HPI** | **PMSH** | **ROS** | **Meds** | **Note** | **Note512** |
| **Random Classifier** | 0.231 (1E-03) | --- | --- | --- | --- | --- | --- | --- | --- |
| **Age & Meds** | 0.365 (2E-03) | --- | --- | --- | --- | --- | --- | --- | --- |
| **Random Forest** | --- | 0.456 (2E-03) | 0.445 (2E-03) | 0.394 (2E-03) | 0.391 (2E-03) | 0.448 (2E-03) | 0.474 (2E-03) | 0.509 (2E-03) | 0.457 (2E-03) |
| **Support Vector Machine** | --- | 0.431 (2E-03) | 0.392 (2E-03) | 0.510 (2E-03) | 0.382 (2E-03) | 0.483 (2E-03) | 0.463 (2E-03) | 0.588 (2E-03) | 0.566 (2E-03) |
| **fastText** | --- | 0.439 (2E-03) | 0.450 (2E-03) | 0.491 (2E-03) | 0.416 (2E-03) | 0.510 (2E-03) | 0.476 (2E-03) | 0.606 (2E-03) | 0.580 (2E-03) |
| **BioClinicalBERT** | --- | 0.441 (2E-03) | 0.453 (2E-03) | 0.545 (2E-03) | 0.400 (2E-03) | 0.530 (2E-03) | 0.496 (2E-03) | 0.600 (2E-03) | 0.590 (2E-03) |

| **B. Class-specific F1** | | | | | | | | | | |
| --- | --- | --- | --- | --- | --- | --- | --- | --- | --- | --- |
|  | | **Baseline** | **Diagnosis** | **Procedure** | **HPI** | **PMSH** | **ROS** | **Meds** | **Note** | **Note512** |
| **Random Classifier** | **I** | 0.133 (2E-03) | --- | --- | --- | --- | --- | --- | --- | --- |
|  | **II** | 0.278 (3E-03) | --- | --- | --- | --- | --- | --- | --- | --- |
|  | **III** | 0.314 (2E-03) | --- | --- | --- | --- | --- | --- | --- | --- |
|  | **IV-V** | 0.199 (3E-03) | --- | --- | --- | --- | --- | --- | --- | --- |
| **Age & Meds** | **I** | 0.413 (3E-03) | --- | --- | --- | --- | --- | --- | --- | --- |
|  | **II** | 0.345 (3E-03) | --- | --- | --- | --- | --- | --- | --- | --- |
|  | **III** | 0.371 (2E-03) | --- | --- | --- | --- | --- | --- | --- | --- |
|  | **IV-V** | 0.329 (3E-03) | --- | --- | --- | --- | --- | --- | --- | --- |
| **Random Forest** | **I** | --- | 0.293 (5E-03) | 0.242 (5E-03) | 0.160 (5E-03) | 0.335 (4E-03) | 0.341 (5E-03) | 0.303 (5E-03) | 0.353 (5E-03) | 0.290 (5E-03) |
|  | **II** | --- | 0.489 (2E-03) | 0.507 (2E-03) | 0.530 (2E-03) | 0.390 (3E-03) | 0.536 (2E-03) | 0.537 (2E-03) | 0.601 (2E-03) | 0.533 (2E-03) |
|  | **III** | --- | 0.560 (2E-03) | 0.581 (2E-03) | 0.644 (2E-03) | 0.593 (2E-03) | 0.629 (2E-03) | 0.628 (2E-03) | 0.647 (2E-03) | 0.656 (2E-03) |
|  | **IV-V** | --- | 0.480 (3E-03) | 0.451 (4E-03) | 0.244 (4E-03) | 0.247 (4E-03) | 0.285 (4E-03) | 0.428 (4E-03) | 0.437 (4E-03) | 0.349 (4E-03) |
| **Support Vector Machine** | **I** | --- | 0.338 (4E-03) | 0.303 (3E-03) | 0.422 (4E-03) | 0.355 (3E-03) | 0.458 (3E-03) | 0.422 (3E-03) | 0.565 (4E-03) | 0.540 (4E-03) |
|  | **II** | --- | 0.415 (3E-03) | 0.379 (3E-03) | 0.538 (2E-03) | 0.343 (3E-03) | 0.445 (3E-03) | 0.428 (3E-03) | 0.608 (2E-03) | 0.574 (2E-03) |
|  | **III** | --- | 0.477 (2E-03) | 0.402 (2E-03) | 0.560 (2E-03) | 0.441 (2E-03) | 0.528 (2E-03) | 0.490 (2E-03) | 0.612 (2E-03) | 0.591 (2E-03) |
|  | **IV-V** | --- | 0.494 (3E-03) | 0.483 (3E-03) | 0.520 (3E-03) | 0.388 (3E-03) | 0.503 (3E-03) | 0.512 (3E-03) | 0.567 (3E-03) | 0.558 (3E-03) |
| **fastText** | **I** | --- | 0.195 (5E-03) | 0.253 (5E-03) | 0.333 (5E-03) | 0.312 (5E-03) | 0.395 (5E-03) | 0.283 (5E-03) | 0.560 (5E-03) | 0.530 (5E-03) |
|  | **II** | --- | 0.525 (2E-03) | 0.509 (2E-03) | 0.558 (2E-03) | 0.473 (2E-03) | 0.584 (2E-03) | 0.543 (2E-03) | 0.633 (2E-03) | 0.613 (2E-03) |
|  | **III** | --- | 0.606 (2E-03) | 0.608 (2E-03) | 0.641 (2E-03) | 0.600 (2E-03) | 0.650 (2E-03) | 0.640 (2E-03) | 0.692 (2E-03) | 0.671 (2E-03) |
|  | **IV-V** | --- | 0.429 (4E-03) | 0.431 (4E-03) | 0.432 (4E-03) | 0.280 (4E-03) | 0.412 (4E-03) | 0.437 (4E-03) | 0.540 (4E-03) | 0.504 (4E-03) |
| **BioClinicalBERT** | **I** | --- | 0.365 (3E-03) | 0.347 (4E-03) | 0.530 (4E-03) | 0.398 (3E-03) | 0.506 (4E-03) | 0.416 (4E-03) | 0.578 (4E-03) | 0.577 (4E-03) |
|  | **II** | --- | 0.422 (3E-03) | 0.475 (2E-03) | 0.561 (2E-03) | 0.384 (3E-03) | 0.532 (2E-03) | 0.507 (3E-03) | 0.607 (2E-03) | 0.611 (2E-03) |
|  | **III** | --- | 0.459 (2E-03) | 0.498 (2E-03) | 0.560 (2E-03) | 0.410 (2E-03) | 0.594 (2E-03) | 0.560 (2E-03) | 0.644 (2E-03) | 0.594 (2E-03) |
|  | **IV-V** | --- | 0.519 (3E-03) | 0.493 (3E-03) | 0.530 (3E-03) | 0.408 (3E-03) | 0.488 (3E-03) | 0.501 (3E-03) | 0.570 (3E-03) | 0.577 (3E-03) |

Supplemental Table 2: (A) Macro-average F1 and (B) class-specific F1 for each model architecture and task on the held-out test set compared to baseline models. Standard errors are reported in parenthesis

###

### Supplemental Table 3: Precision

| **A. Macro-average Precision** | | | | | | | | | |
| --- | --- | --- | --- | --- | --- | --- | --- | --- | --- |
|  | **Baseline** | **Diagnosis** | **Procedure** | **HPI** | **PMSH** | **ROS** | **Meds** | **Note** | **Note512** |
| **Random Classifier** | 0.250 (1E-03) | --- | --- | --- | --- | --- | --- | --- | --- |
| **Age & Meds** | 0.374 (2E-03) | --- | --- | --- | --- | --- | --- | --- | --- |
| **Random Forest** | --- | 0.460 (2E-03) | 0.468 (2E-03) | 0.581 (4E-03) | 0.401 (2E-03) | 0.507 (2E-03) | 0.511 (2E-03) | 0.604 (3E-03) | 0.579 (3E-03) |
| **Support Vector**  **Machine** | --- | 0.426 (2E-03) | 0.420 (1E-03) | 0.501 (2E-03) | 0.389 (2E-03) | 0.483 (2E-03) | 0.462 (1E-03) | 0.573 (2E-03) | 0.550 (2E-03) |
| **fastText** | --- | 0.524 (3E-03) | 0.509 (2E-03) | 0.558 (2E-03) | 0.478 (3E-03) | 0.557 (2E-03) | 0.516 (2E-03) | 0.631 (2E-03) | 0.616 (2E-03) |
| **BioClinicalBERT** | --- | 0.451 (2E-03) | 0.444 (2E-03) | 0.531 (2E-03) | 0.412 (2E-03) | 0.516 (2E-03) | 0.484 (2E-03) | 0.591 (2E-03) | 0.576 (2E-03) |

| **B. Class-specific Precision** | | | | | | | | | | |
| --- | --- | --- | --- | --- | --- | --- | --- | --- | --- | --- |
|  | | **Baseline** | **Diagnosis** | **Procedure** | **HPI** | **PMSH** | **ROS** | **Meds** | **Note** | **Note512** |
| **Random Classifier** | **I** | 0.090 (2E-03) | --- | --- | --- | --- | --- | --- | --- | --- |
|  | **II** | 0.313 (3E-03) | --- | --- | --- | --- | --- | --- | --- | --- |
|  | **III** | 0.431 (3E-03) | --- | --- | --- | --- | --- | --- | --- | --- |
|  | **IV-V** | 0.165 (2E-03) | --- | --- | --- | --- | --- | --- | --- | --- |
| **Age & Meds** | **I** | 0.284 (3E-03) | --- | --- | --- | --- | --- | --- | --- | --- |
|  | **II** | 0.422 (3E-03) | --- | --- | --- | --- | --- | --- | --- | --- |
|  | **III** | 0.538 (3E-03) | --- | --- | --- | --- | --- | --- | --- | --- |
|  | **IV-V** | 0.252 (3E-03) | --- | --- | --- | --- | --- | --- | --- | --- |
| **Random Forest** | **I** | --- | 0.318 (5E-03) | 0.300 (6E-03) | 0.550 (1E-02) | 0.299 (4E-03) | 0.414 (6E-03) | 0.355 (6E-03) | 0.643 (8E-03) | 0.580 (9E-03) |
|  | **II** | --- | 0.493 (3E-03) | 0.487 (3E-03) | 0.512 (3E-03) | 0.457 (3E-03) | 0.498 (3E-03) | 0.509 (3E-03) | 0.534 (3E-03) | 0.529 (3E-03) |
|  | **III** | --- | 0.550 (2E-03) | 0.553 (2E-03) | 0.548 (2E-03) | 0.530 (2E-03) | 0.581 (2E-03) | 0.592 (2E-03) | 0.619 (2E-03) | 0.568 (2E-03) |
|  | **IV-V** | --- | 0.478 (4E-03) | 0.532 (4E-03) | 0.713 (8E-03) | 0.319 (5E-03) | 0.533 (6E-03) | 0.586 (5E-03) | 0.619 (5E-03) | 0.638 (6E-03) |
| **Support Vector Machine** | **I** | --- | 0.248 (3E-03) | 0.194 (2E-03) | 0.394 (5E-03) | 0.234 (3E-03) | 0.315 (3E-03) | 0.294 (3E-03) | 0.508 (5E-03) | 0.471 (4E-03) |
|  | **II** | --- | 0.470 (3E-03) | 0.478 (3E-03) | 0.518 (3E-03) | 0.401 (3E-03) | 0.545 (3E-03) | 0.511 (3E-03) | 0.602 (3E-03) | 0.569 (3E-03) |
|  | **III** | --- | 0.582 (3E-03) | 0.611 (3E-03) | 0.626 (3E-03) | 0.578 (3E-03) | 0.654 (3E-03) | 0.634 (3E-03) | 0.683 (2E-03) | 0.661 (2E-03) |
|  | **IV-V** | --- | 0.405 (3E-03) | 0.398 (3E-03) | 0.465 (4E-03) | 0.343 (3E-03) | 0.416 (3E-03) | 0.410 (3E-03) | 0.499 (3E-03) | 0.498 (3E-03) |
| **fastText** | **I** | --- | 0.474 (1E-02) | 0.414 (8E-03) | 0.543 (8E-03) | 0.427 (7E-03) | 0.511 (6E-03) | 0.379 (6E-03) | 0.625 (6E-03) | 0.625 (6E-03) |
|  | **II** | --- | 0.489 (3E-03) | 0.492 (3E-03) | 0.535 (3E-03) | 0.452 (3E-03) | 0.550 (3E-03) | 0.526 (3E-03) | 0.633 (3E-03) | 0.597 (3E-03) |
|  | **III** | --- | 0.557 (2E-03) | 0.553 (2E-03) | 0.587 (2E-03) | 0.540 (2E-03) | 0.610 (2E-03) | 0.591 (2E-03) | 0.656 (2E-03) | 0.632 (2E-03) |
|  | **IV-V** | --- | 0.576 (5E-03) | 0.576 (5E-03) | 0.568 (5E-03) | 0.492 (6E-03) | 0.556 (5E-03) | 0.569 (5E-03) | 0.609 (4E-03) | 0.611 (4E-03) |
| **BioClinicalBERT** | **I** | --- | 0.243 (2E-03) | 0.289 (4E-03) | 0.469 (5E-03) | 0.274 (3E-03) | 0.382 (4E-03) | 0.328 (4E-03) | 0.547 (5E-03) | 0.521 (5E-03) |
|  | **II** | --- | 0.488 (3E-03) | 0.472 (3E-03) | 0.546 (3E-03) | 0.462 (3E-03) | 0.557 (3E-03) | 0.520 (3E-03) | 0.613 (3E-03) | 0.582 (3E-03) |
|  | **III** | --- | 0.642 (3E-03) | 0.595 (3E-03) | 0.647 (3E-03) | 0.601 (3E-03) | 0.655 (3E-03) | 0.631 (3E-03) | 0.665 (2E-03) | 0.690 (2E-03) |
|  | **IV-V** | --- | 0.432 (3E-03) | 0.419 (3E-03) | 0.462 (3E-03) | 0.312 (3E-03) | 0.471 (4E-03) | 0.456 (4E-03) | 0.537 (4E-03) | 0.510 (3E-03) |

Supplemental Table 3: (A) Macro-average precision and (B) class-specific precision for each model architecture and task on the held-out test set compared to baseline models. Standard errors are reported in parenthesis

###

### Supplemental Table 4: Recall

| **A. Macro-average Recall** | | | | | | | | | |
| --- | --- | --- | --- | --- | --- | --- | --- | --- | --- |
|  | **Baseline** | **Diagnosis** | **Procedure** | **HPI** | **PMSH** | **ROS** | **Meds** | **Note** | **Note512** |
| **Random Classifier** | 0.250 (2E-03) | --- | --- | --- | --- | --- | --- | --- | --- |
| **Age & Meds** | 0.453 (2E-03) | --- | --- | --- | --- | --- | --- | --- | --- |
| **Random Forest** | --- | 0.453 (2E-03) | 0.434 (2E-03) | 0.393 (1E-03) | 0.399 (2E-03) | 0.438 (2E-03) | 0.460 (2E-03) | 0.486 (2E-03) | 0.437 (2E-03) |
| **Support Vector Machine** | --- | 0.486 (2E-03) | 0.482 (2E-03) | 0.527 (2E-03) | 0.459 (2E-03) | 0.572 (2E-03) | 0.548 (2E-03) | 0.615 (2E-03) | 0.596 (2E-03) |
| **fastText** | --- | 0.424 (2E-03) | 0.432 (2E-03) | 0.469 (2E-03) | 0.403 (2E-03) | 0.491 (2E-03) | 0.460 (2E-03) | 0.590 (2E-03) | 0.558 (2E-03) |
| **BioClinicalBERT** | --- | 0.527 (2E-03) | 0.485 (2E-03) | 0.576 (2E-03) | 0.489 (2E-03) | 0.577 (2E-03) | 0.530 (2E-03) | 0.611 (2E-03) | 0.619 (2E-03) |

| **B. Class-specific Recall** | | | | | | | | | | |
| --- | --- | --- | --- | --- | --- | --- | --- | --- | --- | --- |
|  | | **Baseline** | **Diagnosis** | **Procedure** | **HPI** | **PMSH** | **ROS** | **Meds** | **Note** | **Note512** |
| **Random Classifier** | **I** | 0.251 (5E-03) | --- | --- | --- | --- | --- | --- | --- | --- |
|  | **II** | 0.249 (2E-03) | --- | --- | --- | --- | --- | --- | --- | --- |
|  | **III** | 0.247 (2E-03) | --- | --- | --- | --- | --- | --- | --- | --- |
|  | **IV-V** | 0.251 (3E-03) | --- | --- | --- | --- | --- | --- | --- | --- |
| **Age & Meds** | **I** | 0.762 (4E-03) | --- | --- | --- | --- | --- | --- | --- | --- |
|  | **II** | 0.292 (3E-03) | --- | --- | --- | --- | --- | --- | --- | --- |
|  | **III** | 0.284 (2E-03) | --- | --- | --- | --- | --- | --- | --- | --- |
|  | **IV-V** | 0.477 (4E-03) | --- | --- | --- | --- | --- | --- | --- | --- |
| **Random Forest** | **I** | --- | 0.271 (5E-03) | 0.203 (4E-03) | 0.094 (3E-03) | 0.380 (5E-03) | 0.290 (5E-03) | 0.264 (5E-03) | 0.243 (4E-03) | 0.193 (4E-03) |
|  | **II** | --- | 0.486 (3E-03) | 0.528 (3E-03) | 0.550 (3E-03) | 0.340 (3E-03) | 0.581 (3E-03) | 0.569 (3E-03) | 0.687 (3E-03) | 0.537 (3E-03) |
|  | **III** | --- | 0.571 (2E-03) | 0.612 (2E-03) | 0.780 (2E-03) | 0.674 (2E-03) | 0.687 (2E-03) | 0.669 (2E-03) | 0.677 (2E-03) | 0.777 (2E-03) |
|  | **IV-V** | --- | 0.483 (4E-03) | 0.391 (4E-03) | 0.147 (3E-03) | 0.201 (3E-03) | 0.195 (3E-03) | 0.337 (4E-03) | 0.337 (4E-03) | 0.240 (3E-03) |
| **Support Vector Machine** | **I** | --- | 0.532 (5E-03) | 0.700 (5E-03) | 0.454 (5E-03) | 0.736 (5E-03) | 0.834 (4E-03) | 0.746 (5E-03) | 0.637 (5E-03) | 0.633 (5E-03) |
|  | **II** | --- | 0.371 (3E-03) | 0.314 (3E-03) | 0.559 (3E-03) | 0.299 (2E-03) | 0.375 (3E-03) | 0.368 (3E-03) | 0.613 (3E-03) | 0.578 (3E-03) |
|  | **III** | --- | 0.404 (2E-03) | 0.300 (2E-03) | 0.506 (2E-03) | 0.357 (2E-03) | 0.443 (2E-03) | 0.399 (2E-03) | 0.555 (2E-03) | 0.535 (2E-03) |
|  | **IV-V** | --- | 0.636 (4E-03) | 0.613 (4E-03) | 0.588 (4E-03) | 0.446 (4E-03) | 0.637 (4E-03) | 0.679 (4E-03) | 0.657 (4E-03) | 0.636 (4E-03) |
| **fastText** | **I** | --- | 0.123 (4E-03) | 0.182 (4E-03) | 0.240 (4E-03) | 0.246 (5E-03) | 0.322 (5E-03) | 0.226 (4E-03) | 0.508 (5E-03) | 0.461 (5E-03) |
|  | **II** | --- | 0.567 (3E-03) | 0.527 (3E-03) | 0.582 (3E-03) | 0.495 (3E-03) | 0.622 (3E-03) | 0.561 (3E-03) | 0.634 (3E-03) | 0.630 (3E-03) |
|  | **III** | --- | 0.665 (2E-03) | 0.676 (2E-03) | 0.705 (2E-03) | 0.675 (2E-03) | 0.694 (2E-03) | 0.698 (2E-03) | 0.732 (2E-03) | 0.715 (2E-03) |
|  | **IV-V** | --- | 0.342 (4E-03) | 0.344 (4E-03) | 0.349 (4E-03) | 0.195 (3E-03) | 0.327 (4E-03) | 0.355 (4E-03) | 0.485 (4E-03) | 0.429 (4E-03) |
| **BioClinicalBERT** | **I** | --- | 0.729 (5E-03) | 0.436 (5E-03) | 0.610 (5E-03) | 0.724 (5E-03) | 0.748 (4E-03) | 0.568 (5E-03) | 0.611 (5E-03) | 0.647 (5E-03) |
|  | **II** | --- | 0.372 (3E-03) | 0.479 (3E-03) | 0.577 (3E-03) | 0.328 (3E-03) | 0.510 (3E-03) | 0.494 (3E-03) | 0.601 (3E-03) | 0.643 (3E-03) |
|  | **III** | --- | 0.357 (2E-03) | 0.428 (2E-03) | 0.493 (2E-03) | 0.311 (2E-03) | 0.544 (2E-03) | 0.504 (2E-03) | 0.625 (2E-03) | 0.521 (2E-03) |
|  | **IV-V** | --- | 0.651 (4E-03) | 0.599 (4E-03) | 0.621 (4E-03) | 0.593 (4E-03) | 0.505 (4E-03) | 0.555 (4E-03) | 0.607 (4E-03) | 0.664 (4E-03) |

Supplemental Table 4: (A) Macro-average recall and (B) class-specific recall for each model architecture and task on the held-out test set compared to baseline models. Standard errors are reported in parenthesis

### Supplemental Table 5: AUROC

| **A. Macro-average AUROC** | | | | | | | | | |
| --- | --- | --- | --- | --- | --- | --- | --- | --- | --- |
|  | **Baseline** | **Diagnosis** | **Procedure** | **HPI** | **PMSH** | **ROS** | **Meds** | **Note** | **Note512** |
| **Random Classifier** | 0.500 (0E+00) | --- | --- | --- | --- | --- | --- | --- | --- |
| **Age & Meds** | 0.709 (1E-03) | --- | --- | --- | --- | --- | --- | --- | --- |
| **Random Forest** | --- | 0.741 (1E-03) | 0.751 (1E-03) | 0.788 (9E-04) | 0.695 (1E-03) | 0.778 (1E-03) | 0.781 (1E-03) | 0.820 (8E-04) | 0.802 (9E-04) |
| **Support Vector Machine** | --- | 0.714 (1E-03) | 0.717 (1E-03) | 0.789 (1E-03) | 0.697 (1E-03) | 0.787 (9E-04) | 0.768 (9E-04) | 0.850 (8E-04) | 0.829 (9E-04) |
| **fastText** | --- | 0.757 (1E-03) | 0.758 (1E-03) | 0.791 (1E-03) | 0.720 (1E-03) | 0.793 (1E-03) | 0.789 (1E-03) | 0.865 (8E-04) | 0.844 (8E-04) |
| **BioClinicalBERT** | --- | 0.767 (1E-03) | 0.755 (1E-03) | 0.814 (9E-04) | 0.737 (1E-03) | 0.806 (9E-04) | 0.784 (9E-04) | 0.843 (9E-04) | 0.845 (9E-04) |

| **B. Class-specific AUROC** | | | | | | | | | | |
| --- | --- | --- | --- | --- | --- | --- | --- | --- | --- | --- |
|  | | **Baseline** | **Diagnosis** | **Procedure** | **HPI** | **PMSH** | **ROS** | **Meds** | **Note** | **Note512** |
| **Random Classifier** | **I** | 0.500 (0E+00) | --- | --- | --- | --- | --- | --- | --- | --- |
|  | **II** | 0.500 (0E+00) | --- | --- | --- | --- | --- | --- | --- | --- |
|  | **III** | 0.500 (0E+00) | --- | --- | --- | --- | --- | --- | --- | --- |
|  | **IV-V** | 0.500 (0E+00) | --- | --- | --- | --- | --- | --- | --- | --- |
| **Age & Meds** | **I** | 0.863 (2E-03) | --- | --- | --- | --- | --- | --- | --- | --- |
|  | **II** | 0.638 (2E-03) | --- | --- | --- | --- | --- | --- | --- | --- |
|  | **III** | 0.668 (2E-03) | --- | --- | --- | --- | --- | --- | --- | --- |
|  | **IV-V** | 0.668 (2E-03) | --- | --- | --- | --- | --- | --- | --- | --- |
| **Random Forest** | **I** | --- | 0.790 (2E-03) | 0.810 (2E-03) | 0.864 (2E-03) | 0.810 (2E-03) | 0.869 (2E-03) | 0.861 (2E-03) | 0.898 (1E-03) | 0.886 (2E-03) |
|  | **II** | --- | 0.708 (2E-03) | 0.713 (2E-03) | 0.744 (2E-03) | 0.636 (2E-03) | 0.729 (2E-03) | 0.738 (2E-03) | 0.783 (1E-03) | 0.759 (2E-03) |
|  | **III** | --- | 0.660 (2E-03) | 0.674 (2E-03) | 0.708 (2E-03) | 0.644 (2E-03) | 0.708 (2E-03) | 0.718 (2E-03) | 0.747 (1E-03) | 0.719 (2E-03) |
|  | **IV-V** | --- | 0.804 (2E-03) | 0.806 (2E-03) | 0.835 (2E-03) | 0.691 (2E-03) | 0.803 (2E-03) | 0.807 (2E-03) | 0.854 (1E-03) | 0.844 (2E-03) |
| **Support Vector Machine** | **I** | --- | 0.776 (3E-03) | 0.793 (3E-03) | 0.874 (2E-03) | 0.827 (2E-03) | 0.904 (1E-03) | 0.869 (2E-03) | 0.938 (1E-03) | 0.924 (1E-03) |
|  | **II** | --- | 0.652 (2E-03) | 0.632 (2E-03) | 0.738 (2E-03) | 0.592 (2E-03) | 0.691 (2E-03) | 0.679 (2E-03) | 0.806 (1E-03) | 0.775 (1E-03) |
|  | **III** | --- | 0.638 (2E-03) | 0.649 (2E-03) | 0.709 (2E-03) | 0.655 (2E-03) | 0.728 (2E-03) | 0.702 (2E-03) | 0.775 (1E-03) | 0.750 (2E-03) |
|  | **IV-V** | --- | 0.789 (2E-03) | 0.794 (2E-03) | 0.836 (2E-03) | 0.714 (2E-03) | 0.826 (2E-03) | 0.821 (2E-03) | 0.881 (1E-03) | 0.865 (1E-03) |
| **fastText** | **I** | --- | 0.815 (2E-03) | 0.820 (2E-03) | 0.870 (2E-03) | 0.833 (2E-03) | 0.889 (2E-03) | 0.863 (2E-03) | 0.943 (1E-03) | 0.930 (1E-03) |
|  | **II** | --- | 0.724 (2E-03) | 0.718 (2E-03) | 0.755 (2E-03) | 0.675 (2E-03) | 0.771 (2E-03) | 0.755 (2E-03) | 0.833 (1E-03) | 0.809 (1E-03) |
|  | **III** | --- | 0.684 (2E-03) | 0.685 (2E-03) | 0.720 (2E-03) | 0.668 (2E-03) | 0.729 (2E-03) | 0.724 (2E-03) | 0.798 (1E-03) | 0.771 (1E-03) |
|  | **IV-V** | --- | 0.805 (2E-03) | 0.811 (2E-03) | 0.819 (2E-03) | 0.702 (2E-03) | 0.782 (2E-03) | 0.815 (2E-03) | 0.884 (1E-03) | 0.867 (1E-03) |
| **BioClinicalBERT** | **I** | --- | 0.838 (2E-03) | 0.816 (2E-03) | 0.901 (2E-03) | 0.851 (2E-03) | 0.902 (1E-03) | 0.861 (2E-03) | 0.917 (2E-03) | 0.922 (1E-03) |
|  | **II** | --- | 0.711 (2E-03) | 0.707 (2E-03) | 0.768 (2E-03) | 0.674 (2E-03) | 0.748 (2E-03) | 0.737 (2E-03) | 0.806 (1E-03) | 0.804 (1E-03) |
|  | **III** | --- | 0.688 (2E-03) | 0.681 (2E-03) | 0.741 (2E-03) | 0.682 (2E-03) | 0.752 (2E-03) | 0.719 (2E-03) | 0.776 (1E-03) | 0.779 (1E-03) |
|  | **IV-V** | --- | 0.830 (2E-03) | 0.818 (2E-03) | 0.848 (2E-03) | 0.741 (2E-03) | 0.823 (2E-03) | 0.818 (2E-03) | 0.874 (1E-03) | 0.874 (1E-03) |

Supplemental Table 5: (A) Macro-average AUROC and (B) class-specific AUROC for each model architecture and task on the held-out test set compared to baseline models. Standard errors are reported in parenthesis

### Supplemental Table 6: AUPRC

| **A. Macro-average AUPRC** | | | | | | | | | |
| --- | --- | --- | --- | --- | --- | --- | --- | --- | --- |
|  | **Baseline** | **Diagnosis** | **Procedure** | **HPI** | **PMSH** | **ROS** | **Meds** | **Note** | **Note512** |
| **Random Classifier** | 0.250 (4E-09) | --- | --- | --- | --- | --- | --- | --- | --- |
| **Age & Meds** | 0.416 (2E-03) | --- | --- | --- | --- | --- | --- | --- | --- |
| **Random Forest** | --- | 0.457 (2E-03) | 0.462 (2E-03) | 0.510 (2E-03) | 0.392 (2E-03) | 0.484 (2E-03) | 0.489 (2E-03) | 0.567 (2E-03) | 0.534 (2E-03) |
| **Support Vector Machine** | --- | 0.444 (2E-03) | 0.451 (2E-03) | 0.525 (2E-03) | 0.413 (2E-03) | 0.514 (2E-03) | 0.490 (2E-03) | 0.627 (2E-03) | 0.593 (2E-03) |
| **fastText** | --- | 0.478 (2E-03) | 0.473 (2E-03) | 0.518 (2E-03) | 0.421 (2E-03) | 0.512 (2E-03) | 0.495 (2E-03) | 0.642 (2E-03) | 0.607 (2E-03) |
| **BioClinicalBERT** | --- | 0.486 (2E-03) | 0.473 (2E-03) | 0.570 (2E-03) | 0.446 (2E-03) | 0.537 (2E-03) | 0.499 (2E-03) | 0.617 (2E-03) | 0.620 (2E-03) |

| **B. Class-specific AUPRC** | | | | | | | | | | |
| --- | --- | --- | --- | --- | --- | --- | --- | --- | --- | --- |
|  | | **Baseline** | **Diagnosis** | **Procedure** | **HPI** | **PMSH** | **ROS** | **Meds** | **Note** | **Note512** |
| **Random Classifier** | **I** | 0.091 (9E-04) | --- | --- | --- | --- | --- | --- | --- | --- |
|  | **II** | 0.316 (1E-03) | --- | --- | --- | --- | --- | --- | --- | --- |
|  | **III** | 0.429 (2E-03) | --- | --- | --- | --- | --- | --- | --- | --- |
|  | **IV-V** | 0.163 (1E-03) | --- | --- | --- | --- | --- | --- | --- | --- |
| **Age & Meds** | **I** | 0.384 (5E-03) | --- | --- | --- | --- | --- | --- | --- | --- |
|  | **II** | 0.425 (3E-03) | --- | --- | --- | --- | --- | --- | --- | --- |
|  | **III** | 0.568 (3E-03) | --- | --- | --- | --- | --- | --- | --- | --- |
|  | **IV-V** | 0.289 (3E-03) | --- | --- | --- | --- | --- | --- | --- | --- |
| **Random Forest** | **I** | --- | 0.285 (5E-03) | 0.285 (4E-03) | 0.394 (6E-03) | 0.295 (4E-03) | 0.374 (5E-03) | 0.327 (4E-03) | 0.488 (6E-03) | 0.455 (6E-03) |
|  | **II** | --- | 0.490 (3E-03) | 0.487 (3E-03) | 0.518 (3E-03) | 0.425 (3E-03) | 0.515 (3E-03) | 0.498 (3E-03) | 0.580 (3E-03) | 0.550 (3E-03) |
|  | **III** | --- | 0.565 (3E-03) | 0.576 (3E-03) | 0.614 (3E-03) | 0.551 (3E-03) | 0.610 (2E-03) | 0.621 (3E-03) | 0.650 (3E-03) | 0.625 (3E-03) |
|  | **IV-V** | --- | 0.488 (4E-03) | 0.500 (4E-03) | 0.514 (4E-03) | 0.299 (3E-03) | 0.437 (4E-03) | 0.510 (4E-03) | 0.550 (4E-03) | 0.508 (4E-03) |
| **Support Vector Machine** | **I** | --- | 0.274 (4E-03) | 0.305 (5E-03) | 0.436 (6E-03) | 0.323 (5E-03) | 0.433 (5E-03) | 0.345 (5E-03) | 0.606 (6E-03) | 0.575 (6E-03) |
|  | **II** | --- | 0.460 (3E-03) | 0.441 (3E-03) | 0.519 (3E-03) | 0.392 (3E-03) | 0.493 (3E-03) | 0.477 (3E-03) | 0.614 (3E-03) | 0.574 (3E-03) |
|  | **III** | --- | 0.568 (3E-03) | 0.566 (2E-03) | 0.618 (3E-03) | 0.570 (3E-03) | 0.639 (3E-03) | 0.618 (3E-03) | 0.684 (3E-03) | 0.655 (3E-03) |
|  | **IV-V** | --- | 0.473 (4E-03) | 0.492 (4E-03) | 0.527 (4E-03) | 0.367 (4E-03) | 0.491 (4E-03) | 0.519 (4E-03) | 0.605 (4E-03) | 0.568 (4E-03) |
| **fastText** | **I** | --- | 0.317 (5E-03) | 0.308 (5E-03) | 0.428 (6E-03) | 0.316 (4E-03) | 0.429 (5E-03) | 0.340 (5E-03) | 0.617 (6E-03) | 0.575 (6E-03) |
|  | **II** | --- | 0.507 (3E-03) | 0.491 (3E-03) | 0.531 (3E-03) | 0.453 (3E-03) | 0.559 (3E-03) | 0.517 (3E-03) | 0.645 (3E-03) | 0.605 (3E-03) |
|  | **III** | --- | 0.590 (3E-03) | 0.583 (3E-03) | 0.620 (3E-03) | 0.568 (3E-03) | 0.617 (3E-03) | 0.622 (3E-03) | 0.705 (3E-03) | 0.675 (3E-03) |
|  | **IV-V** | --- | 0.495 (4E-03) | 0.510 (4E-03) | 0.491 (4E-03) | 0.349 (4E-03) | 0.444 (4E-03) | 0.502 (4E-03) | 0.601 (4E-03) | 0.575 (4E-03) |
| **BioClinicalBERT** | **I** | --- | 0.330 (5E-03) | 0.301 (5E-03) | 0.529 (6E-03) | 0.354 (5E-03) | 0.446 (6E-03) | 0.337 (5E-03) | 0.583 (6E-03) | 0.592 (6E-03) |
|  | **II** | --- | 0.499 (3E-03) | 0.487 (3E-03) | 0.562 (3E-03) | 0.454 (3E-03) | 0.553 (3E-03) | 0.522 (3E-03) | 0.616 (3E-03) | 0.612 (3E-03) |
|  | **III** | --- | 0.599 (3E-03) | 0.585 (3E-03) | 0.641 (3E-03) | 0.588 (3E-03) | 0.655 (2E-03) | 0.628 (3E-03) | 0.680 (3E-03) | 0.690 (3E-03) |
|  | **IV-V** | --- | 0.517 (4E-03) | 0.519 (4E-03) | 0.547 (4E-03) | 0.389 (4E-03) | 0.493 (4E-03) | 0.509 (4E-03) | 0.588 (4E-03) | 0.585 (4E-03) |

Supplemental Table 6: (A) Macro-average AUPRC and (B) class-specific AUPRC for each model architecture and task on the held-out test set compared to baseline models. Standard errors are reported in parenthesis

### Supplemental Table 7: Not Statistically Significant Pairwise Metric Comparisons

| **Metric** | **Model-Task 1** | **Model-Task2** | **P-Value** |
| --- | --- | --- | --- |
| AUPRC/III | ('baseline', 'age_meds') | ('diagnosis', 'svm') | 0.38637871 |
| AUPRC/III | ('baseline', 'age_meds') | ('pmsh', 'fasttext') | 0.571156937 |
| AUPRC/III | ('diagnosis', 'svm') | ('pmsh', 'fasttext') | 0.769673643 |
| AUPRC/III | ('note512', 'svm') | ('ros', 'bioclinicalbert') | 0.109042336 |
| AUPRC/III | ('ros', 'fasttext') | ('hpi', 'svm') | 0.38637871 |
| AUPRC/IV-V | ('procedure', 'bioclinicalbert') | ('meds', 'svm') | 0.097775687 |
| AUPRC/IV-V | ('procedure', 'fasttext') | ('meds', 'bioclinicalbert') | 0.01061713 |
| AUPRC/IV-V | ('procedure', 'fasttext') | ('meds', 'rf') | 0.235042399 |
| AUPRC/IV-V | ('procedure', 'svm') | ('hpi', 'fasttext') | 0.099719727 |
| AUPRC/IV-V | ('ros', 'svm') | ('hpi', 'fasttext') | 0.01582148 |
| AUROC/I | ('meds', 'svm') | ('hpi', 'fasttext') | 0.141642772 |
| AUROC/I | ('meds', 'svm') | ('ros', 'rf') | 0.049022623 |
| AUROC/II | ('meds', 'rf') | ('hpi', 'svm') | 0.330881168 |
| AUROC/III | ('baseline', 'age_meds') | ('pmsh', 'fasttext') | 0.402454357 |
| AUROC/III | ('note512', 'rf') | ('meds', 'bioclinicalbert') | 0.024126655 |
| F1/I | ('note512', 'fasttext') | ('hpi', 'bioclinicalbert') | 0.873680726 |
| F1/III | ('meds', 'bioclinicalbert') | ('diagnosis', 'rf') | 0.48698999 |
| F1/IV-V | ('note512', 'fasttext') | ('ros', 'svm') | 0.83670703 |
| MCC/MCC | ('note', 'rf') | ('hpi', 'bioclinicalbert') | 0.692911628 |
| Precision/Macro | ('meds', 'fasttext') | ('ros', 'bioclinicalbert') | 0.283178459 |

Supplemental Table 7: P-values for all pairwise comparisons which were not statistically significant. Reported p-values are corrected for multiple hypothesis testing using the Benjamini-Hochberg procedure with α=0.01.
